# Behavior of reinforced concrete two-way slabs reinforced with carbon FRP bars under flexural loads

**DOI:** 10.1038/s41598-026-62553-9

**Published:** 2026-07-28

**Authors:** Reham A. Mohamed, Ali S. Shanour, Taha A. El-Sayed

**Affiliations:** Structural Engineering Department, Faculty of Engineering at Shoubra, Benha University, Cairo, Egypt

**Keywords:** Two-way slabs, CFRP reinforcement, HSC slabs, Flexural behavior, ABAQUS, Nonlinear FE analysis, Crack pattern, Engineering, Materials science

## Abstract

This study presents an experimental, numerical, and analytical investigation of the flexural behavior of two-way high-strength concrete (HSC) slabs reinforced with carbon fiber-reinforced polymer (CFRP) bars. Seven slabs with dimensions of 1500 mm × 1500 mm were tested, including one steel-reinforced reference slab and six CFRP-reinforced slabs with two thicknesses, 100 and 120 mm, and three CFRP reinforcement ratios. For the 100 mm slabs, increasing the CFRP reinforcement ratio increased the ultimate load by 13.8% and 24.3% relative to S100-CFRP1, while the corresponding increases for the 120 mm slabs were 31.1% and 48.0% relative to S120-CFRP1. Increasing the slab thickness from 100 to 120 mm enhanced the ultimate load by 27.7%, 47.2%, and 52.1% for the corresponding CFRP-reinforced specimens. A mechanically normalized comparison between the steel-reinforced reference slab and the CFRP-reinforced slab with the same nominal reinforcement ratio showed that the two specimens were not mechanically equivalent. The CFRP-reinforced slab had lower effective reinforcement stiffness but a higher strength-based mechanical reinforcement index, resulting in a higher ultimate load but lower deformation capacity. Crack observations confirmed flexural failure with more localized cracking in the CFRP-reinforced slabs. A nonlinear finite element model was developed in ABAQUS and validated against the experimental results, with an average ultimate-load prediction error of approximately 7.2%. Analytical predictions based on ECP 208–2019 and ACI 440.1R-15 showed average predicted-to-experimental ultimate-load ratios of 1.43 and 1.27, respectively, indicating that ACI 440.1R-15 provided closer estimates than ECP 208–2019 for the tested CFRP-reinforced slabs.

## Introduction

The use of fiber-reinforced polymer (FRP) reinforcement in concrete structures has increased because of its corrosion resistance, high tensile strength, and low self-weight. Although steel reinforcement is widely used, corrosion can reduce durability and increase maintenance costs, especially in aggressive environments. However, FRP bars cannot be treated as a direct replacement for steel because they behave linearly up to rupture without yielding^1,2^. Therefore, FRP-reinforced concrete members should be evaluated in terms of cracking, stiffness, deformation capacity, bond behavior, serviceability, ultimate strength, and failure mode^3,4^.

Several studies investigated FRP-reinforced concrete beams. Barris et al. and Kara et al. showed that the low elastic modulus of GFRP bars strongly affects deflection and crack width^5,6^. CFRP-reinforced beams were also examined because CFRP bars have higher tensile strength and stiffness than GFRP and BFRP bars, although their failure response remains brittle^7,8^. Bond and anchorage conditions were also found to influence crack spacing, strain distribution, and load-deflection behavior^9,10^. El-Sayed and co-workers investigated GFRP-reinforced geopolymer concrete, high-strength concrete members, deep beams, and hybrid GFRP-reinforced HSC beams, showing that concrete type, reinforcement ratio, member geometry, hybridization, and crack propagation affect flexural response and ductility^11–13^. Shear studies also confirmed that FRP modulus, bond behavior, shear span-to-depth ratio, and crack kinematics affect shear capacity and crack development^14–16^. FRP reinforcement has also been applied in columns and walls, where axial stiffness, confinement, temperature exposure, eccentric loading, and member type significantly affect structural behavior^17–19^.

For slab systems, FRP reinforcement has been investigated in one-way and two-way action. Studies on two-way slabs reinforced with FRP bars and grids showed that slab thickness, concrete strength, reinforcement stiffness, support conditions, and FRP bond characteristics affect punching resistance and prediction accuracy^20–22^. Shill et al. investigated large-scale two-way slabs reinforced with CFRP and BFRP rebars and reported different load-deflection behavior, cracking response, ductility, and failure modes compared with steel-reinforced slabs^23^. However, their study mainly considered normal-strength concrete and punching/shear-related response rather than the flexural behavior of CFRP-reinforced HSC two-way slabs.

Externally strengthened two-way slabs using CFRP systems have also been studied. Ebead and Marzouk reported that carbon FRP strips and glass FRP laminates increased flexural capacity, although ductility could decrease^24^. Limam et al. showed that CFRP strips enhanced load-carrying capacity, while debonding remained critical^25^. Torabian and Mostofinejad and Sharhan et al. also showed that grooving techniques and CFRP laminates can improve ultimate load, cracking load, and debonding resistance^26,27^. Nevertheless, these studies mainly focused on strengthening existing slabs rather than newly cast slabs internally reinforced with CFRP bars. In slab-related studies, Erfan et al., Adam et al., and El-Sayed and co-workers showed that FRP reinforcement ratio, material composition, geometry, and reinforcement stiffness influence cracking, deformation, stiffness, and flexural behavior of HSC, porous, and geopolymer slabs^28–30^.

Among FRP materials, CFRP bars are attractive because of their higher tensile strength and stiffness. Yoo et al. investigated ribbed CFRP bars in UHPFRC beams with lap-splice connections and showed that bond and splice performance are essential for flexural response^31^. Yoo and Yoon reported that elevated temperature can reduce the flexural performance of spliced CFRP bars in UHPFRC beams^32^. Further studies by Yoo and co-workers showed that hybrid CFRP-steel reinforcement, post-heating behavior, and anchorage methods significantly affect residual capacity, bond condition, load transfer, and structural performance^33–35^.

Despite the growing body of research on FRP-reinforced concrete members, three specific research gaps remain insufficiently addressed. First, most available studies on slab systems have focused on externally strengthened slabs using CFRP laminates or strips^24–27^, punching/shear-related response of FRP-reinforced two-way slabs^20–23^, or slab systems with different concrete types and reinforcement configurations^28–30^. In contrast, the flexural behavior of newly cast two-way HSC slabs internally reinforced with CFRP bars remains limited. Second, although previous studies have investigated CFRP bars in beams, UHPFRC members, lap-splice systems, and hybrid CFRP–steel reinforcement^31–35^, the combined influence of slab thickness and CFRP reinforcement ratio on the complete flexural response of CFRP-reinforced HSC two-way slabs has not been systematically clarified. This includes ultimate load, deflection, crack localization, toughness, deformability, stiffness degradation, and failure mode. Third, the applicability of available design provisions has not been sufficiently assessed for predicting the flexural strength of this specific slab configuration, particularly with respect to ECP 208-2019^36^ and ACI 440.1R-15^1^.

The present study addresses these gaps through an integrated experimental, numerical, and analytical investigation of CFRP-reinforced HSC two-way slabs under flexural loading. The main contribution is the mechanistic interpretation of the flexural response using mechanically normalized steel–CFRP comparison, reinforcement stiffness, strength-based mechanical reinforcement index, crack localization, deformability, toughness, global secant stiffness, and failure mode. In addition, the validated nonlinear finite element model and the ECP/ACI code comparison provide a design-assessment framework for evaluating the applicability of existing design provisions to this slab configuration.

## Experimental program

The experimental program was conducted in the Reinforced Concrete Laboratory, Faculty of Engineering, Cairo University, Giza, Egypt. The study aimed to investigate the structural performance of high-strength concrete (HSC) slabs reinforced with carbon fiber-reinforced polymer (CFRP) bars under flexural loading.

The behavior of the tested slabs was evaluated in terms of ultimate load, ultimate deflection, concrete and CFRP bar strains, deformability index, stiffness, and crack patterns. Details of the specimens, reinforcement configuration, and testing setup are presented in the following sections.

### Properties of materials

#### Concrete mix design

A concrete mix with a target compressive strength of 75 MPa at 28 days was used in this study. The proportions of the constituent materials are summarized in Table 1**.** The water-to-binder ratio, calculated as water/ (cement + silica fume), was 0.27, while the fine aggregate-to-total aggregate ratio was 0.40. Concrete cubes were cast during the slab casting to determine the compressive strength.

**Table 1:** Concrete mix design.

Materials	Per m^3^ of concrete (*f*_*cu*_ = 75MPa)
Cement (CEM I)	500 kg/m^3^
Coarse aggregate	1080 kg/m^3^
Fine aggregate	720 kg/m^3^
Silica fume (SF)	50 kg/m^3^
Super plasticizers (Auramix340)	14 kg/m^3^
Water	150 kg/m^3^
Water/(Cement + SF) ratio	0.27
Fine agg./ total agg. ratio	0.4

#### Results of compressive strength test

Concrete cubes with dimensions of 150 × 150 × 150 mm were tested after 28 days using a universal testing machine with a capacity of 2000 kN to determine the compressive strength in accordance with ECP 2018^37^. The average 28-day cube compressive strength was 78.3 MPa, with a standard deviation of approximately 4.6 MPa, confirming that the concrete achieved the target high-strength level.

#### Carbon Fiber Reinforced Polymer (CFRP) bars

Carbon fiber-reinforced polymer (CFRP) bars exhibit high tensile strength and stiffness compared to conventional steel reinforcement, making them suitable for high-performance structural applications. In this study, CFRP bars with an ultimate tensile strength of approximately 1400 MPa were used.

**Fig. 1:**
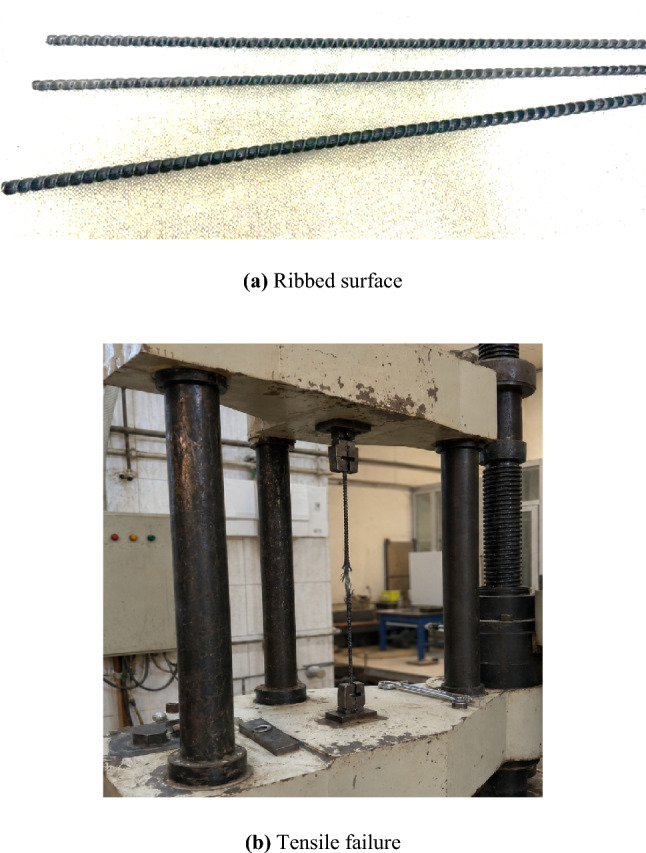
CFRP bars used in the experimental program: (**a**) ribbed surface geometry showing the helical ribs used to improve mechanical interlock with the surrounding concrete; (**b**) tensile failure mode of the CFRP bar after testing.

Figure 1(a) illustrates the surface characteristics of the CFRP bars used in this study, highlighting the surface texture that enhances the bond with the surrounding concrete. Tensile testing was conducted at the National Research Centre (NRC) in accordance with ECP 2018^37^. To minimize the risk of premature failure at the gripping region, the ends of the CFRP bars were reinforced using thin‑walled steel sleeves filled with high‑strength epoxy resin prior to testing. This technique ensures uniform distribution of the clamping forces and prevents local crushing or slip at the grips. The tensile tests were conducted following the recommendations of ECP 2018^37^ and the general principles of ASTM D7205. Only specimens that failed within the free length (middle third of the gauge length) were considered valid for determining the mechanical properties; any specimen that failed at or within one bar diameter of the grip was discarded. As shown in Fig. 1(b)**,** the valid failure mode typically consisted of explosive rupture of the carbon fibers in the central region, confirming that the gripping reinforcement effectively shifted the failure away from the stress‑concentrated end zones.

The CFRP bars used in this study had an ultimate tensile strength of approximately 1400 MPa, an elastic modulus of 150 GPa, and an ultimate tensile strain of about 0.9%. These values confirm the high tensile strength and linear-elastic brittle behavior of CFRP reinforcement. The bars had a ribbed surface configuration, which was used to enhance mechanical interlock with the surrounding concrete.

### Details of tested slabs

Seven two-way slab specimens with plan dimensions of 1500 mm × 1500 mm were cast and tested under flexural loading in this study. The slab thickness varied between 100 and 120 mm. All specimens were made of high-strength concrete with a compressive strength of 75 MPa and a constant concrete cover of 25 mm.

The specimens were divided into three groups. The first group consisted of one control slab reinforced with conventional steel bars (S100-Steel) with a thickness of 100 mm.

The second group (Group A) included three slabs (S100-CFRP1, S100-CFRP2, and S100-CFRP3) reinforced with CFRP bars, all with a thickness of 100 mm and different reinforcement ratios.

**Fig. 2:**
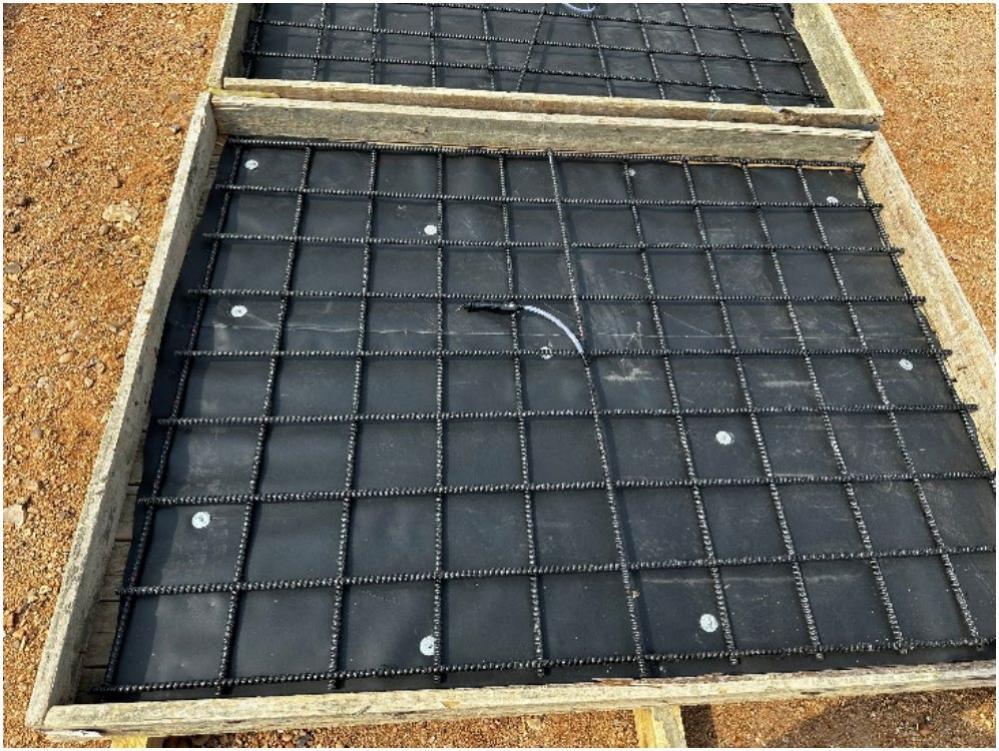
Construction of the tested specimens.

The third group (Group B) consisted of three slabs (S120-CFRP1, S120-CFRP2, and S120-CFRP3) reinforced with CFRP bars, with a thickness of 120 mm and different reinforcement ratios. Details of all tested specimens are presented in Table 2 and Fig. 2**.**

**Table 2:** Details of tested slabs.

Group	Slab ID	*t* (mm)	Bars/m	*Ø* (mm)	*A*_*s*_ (mm^2^/m)	*ρ* (%)	Type
Control	S100-Steel	100	6	8	301.59	0.42	Steel
Group A	S100-CFRP1	100	5	8	251.33	0.35	CFRP
	S100-CFRP2	100	6	8	301.59	0.42	CFRP
	S100-CFRP3	100	7	8	351.86	0.50	CFRP
Group B	S120-CFRP1	120	5	8	251.33	0.28	CFRP
	S120-CFRP2	120	6	8	301.59	0.33	CFRP
	S120-CFRP3	120	7	8	351.86	0.39	CFRP

Three main variables were investigated to evaluate their effect on the flexural behavior of the slabs:

1. Reinforcement ratio (spacing).
2. Slab thickness.
3. Type of reinforcement (CFRP versus steel bars).

where *t* is the slab thickness, *Ø* is the bar diameter, *A*_*s*_ is the reinforcement area per meter width, *ρ* is the reinforcement ratio, *b* is the unit slab width, and d is the effective depth. $\rho =\frac{{A}_{s}}{bd}\times 100$.

### Experimental setup

A rigid steel frame available in the Reinforced Concrete Laboratory, Faculty of Engineering, Cairo University, was SeeFig. 3 used to test the slabs. The applied load from the hydraulic jack was measured using a load cell, as shown in Fig. 4**.**

**Fig. 3:**
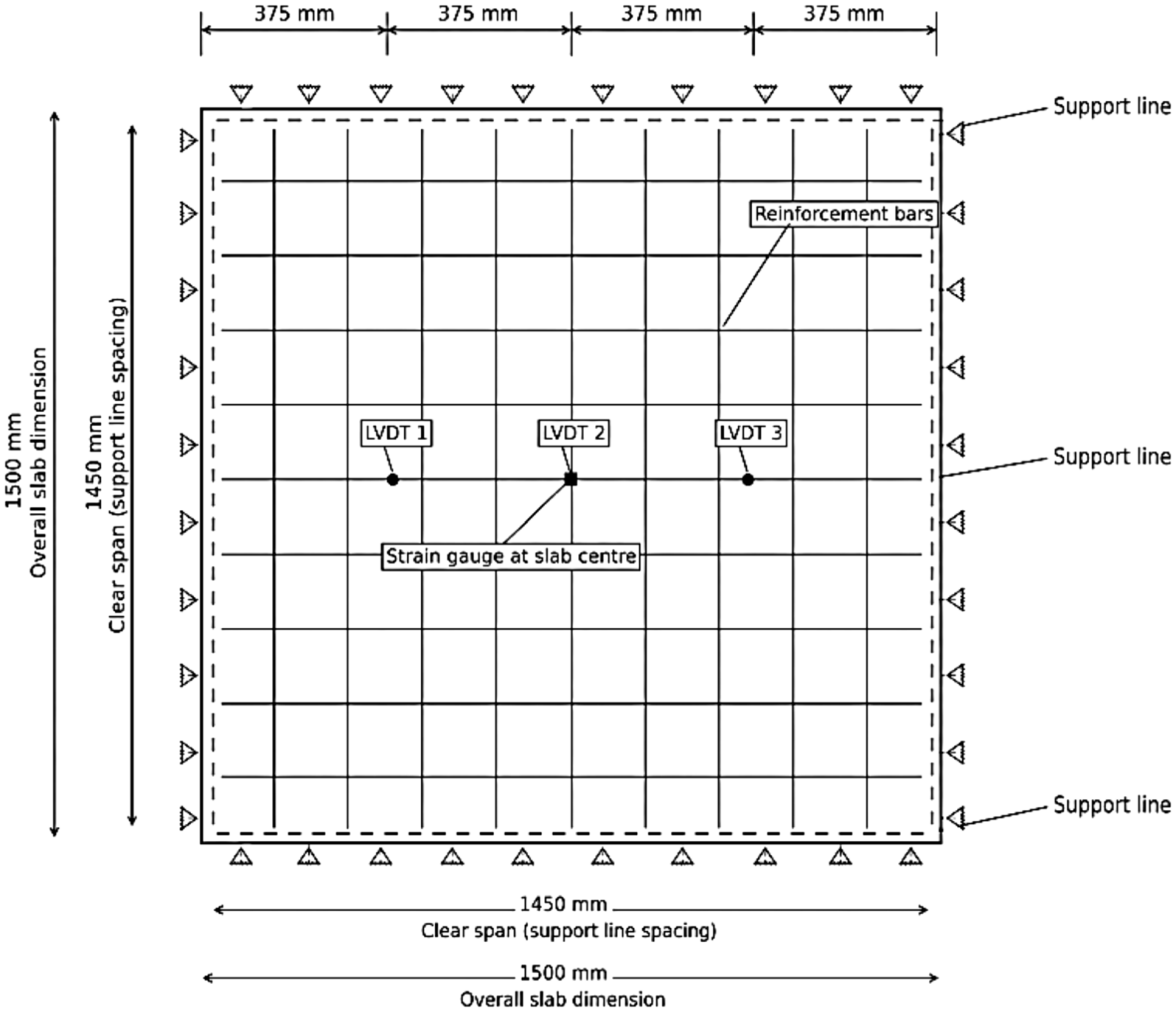
Reinforcement layout, slab dimensions, clear span, support-line locations, LVDT positions, and strain gauge location of the tested slab.

**Fig. 4:**
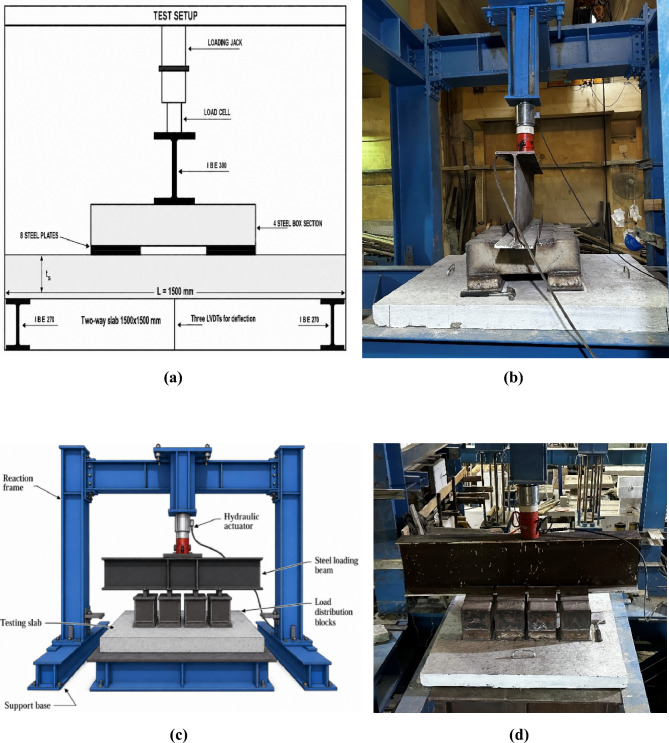
Experimental test setup: (**a**) schematic side view; (**b**) actual side view; (**c**) schematic front view; (**d**) actual front view of the slab specimen during loading.

The strain in the reinforcement was monitored using one strain gauge attached to the surface of the central reinforcement bar at the mid-span region of each tested slab. The strain gauge location is shown in Fig. 3. The vertical displacements were recorded using three linear variable differential transformers (LVDTs) placed beneath the slab at the locations shown in Fig. 3**.** Crack development was visually observed and marked at each loading step. In addition, the applied load and the corresponding deflections were recorded throughout the testing process.

## Test results and discussion

This section presents the load–deflection behavior, ultimate load, ultimate deflection, toughness, deformability index, and secant stiffness of the tested slabs.

### Ultimate load

The ultimate load values of all tested slabs are presented in Table 5**.** The control slab (S100–Steel) exhibited an ultimate load of 249.10 kN, which can be attributed to the ductile behavior of steel reinforcement and its strong bond with the surrounding concrete. This observation is consistent with the findings reported by Janus et al.^38^.

For Group A, slab S100-CFRP2 exhibited an ultimate load of 281.65 kN, which is approximately 13% higher than that of the steel-reinforced control slab S100-Steel. However, this comparison should not be interpreted directly on the basis of nominal reinforcement ratio alone, because steel and CFRP bars have different elastic moduli, tensile strengths, bond characteristics, and stress–strain responses.

Therefore, the comparison between S100-Steel and S100-CFRP2 was mechanically normalized using the mechanical reinforcement index, ${\rho}_{h,f}$ and the effective reinforcement stiffness, ${\rho}_{h,s}$, following the approach adopted by Abbas et al.^39^ for steel–FRP reinforced members. These parameters were calculated using Eqs. (1) and (2), respectively:

1 $${\rho}_{h,f}={\rho}_{s}\frac{{f}_{y}}{{f}_{fu}}+{\rho}_{f}$$

2 $${\rho}_{h,s}={\rho}_{s}+{\rho}_{f}\frac{{E}_{f}}{{E}_{s}}$$

where ${\rho}_{s}$ and ${\rho}_{f}$ are the steel and CFRP reinforcement ratios, respectively; ${f}_{y}$ is the yield stress of steel reinforcement; ${f}_{fu}$ is the ultimate tensile strength of CFRP reinforcement; and ${E}_{s}$ and ${E}_{f}$ are the elastic moduli of steel and CFRP reinforcement, respectively. The mechanical reinforcement index, ${\rho}_{h,f}$, represents the equivalent FRP reinforcement ratio in terms of strength, while the effective reinforcement stiffness, ${\rho}_{h,s}$*,* represents the equivalent steel reinforcement ratio in terms of stiffness.

The results in Table 3 show that S100-CFRP2 had lower effective reinforcement stiffness than S100-Steel due to the lower elastic modulus of CFRP bars, but a higher mechanical reinforcement index due to the high tensile strength of CFRP reinforcement. Therefore, the approximately 13% higher ultimate load of S100-CFRP2 should be attributed to the higher strength-based reinforcement index and post-cracking slab-system response rather than to the nominal reinforcement ratio alone.

**Table 3:** Mechanical normalization of steel and CFRP reinforcement for specimens with the same nominal reinforcement ratio.

**Slab ID**	**Reinforcement type**	${{\boldsymbol{\rho}}}_{{\boldsymbol{s}}}$**(%)**	${{\boldsymbol{\rho}}}_{{\boldsymbol{f}}}$**(%)**	${{\boldsymbol{\rho}}}_{{\boldsymbol{h}},{\boldsymbol{f}}}$**(%)**	${{\boldsymbol{\rho}}}_{{\boldsymbol{h}},{\boldsymbol{s}}}$**(%)**	${\mathbf{P}}_{\mathbf{u}}$**(kN)**	**Mechanical observation**
S100-Steel	Steel	0.42	0.00	0.126	0.420	249.10	Higher effective reinforcement stiffness
S100-CFRP2	CFRP	0.00	0.42	0.420	0.315	281.65	Higher strength-based mechanical reinforcement index

Note: The mechanical normalization values were calculated using ${f}_{y}$= 420 MPa, ${f}_{fu}$= 1400 MPa, ${E}_{s}$= 200 GPa, and ${E}_{f}$= 150 GPa.

Within Group A, slabs S100-CFRP2 and S100-CFRP3 showed increases of 13.8% and 24.3%, respectively, relative to S100-CFRP1 (247.43 kN). This improvement is attributed to the increase in CFRP reinforcement ratio, which enhances the flexural capacity of the slabs, in agreement with Elgabbas et al.^40^.

For Group B, a similar trend was observed but with higher ultimate load capacities. Relative to S120-CFRP1 (316.14 kN), slabs S120-CFRP2 and S120-CFRP3 showed increases of 31.1% and 48.0%, respectively, reflecting the combined effect of increasing both reinforcement ratio and slab thickness.

As expected from flexural mechanics, increasing slab thickness from 100 to 120 mm enhanced the ultimate load of the CFRP-reinforced slabs by 27.7%, 47.2%, and 52.1% for the corresponding reinforcement configurations. This improvement is mainly attributed to the increase in effective depth, flexural rigidity, and internal lever arm, which allows the slab to develop higher resisting moment before failure. This trend is consistent with the observations reported by Adam et al.^29^.

### Ultimate deflection

As shown in Table 5**,** slab S100-CFRP2 exhibited a significantly lower ultimate deflection of 18.16 mm compared to the control slab S100-Steel, which recorded 36.91 mm, representing a reduction of approximately 50.8%.

For Group A (100 mm thickness), slabs S100-CFRP1, S100-CFRP2, and S100-CFRP3 recorded ultimate deflections of 19.47 mm, 18.16 mm, and 17.49 mm, respectively. This reduction in deflection is mainly attributed to the increase in CFRP reinforcement ratio.

For Group B (120 mm thickness), slabs S120-CFRP1, S120-CFRP2, and S120-CFRP3 exhibited lower ultimate deflections of 16.64 mm, 14.13 mm, and 13.20 mm, respectively, which is consistent with Achillides and Pilakoutas^9^.

Compared to Group A, slabs in Group B showed further reductions in deflection due to the combined effect of increased slab thickness and reinforcement ratio.

**Fig. 5:**
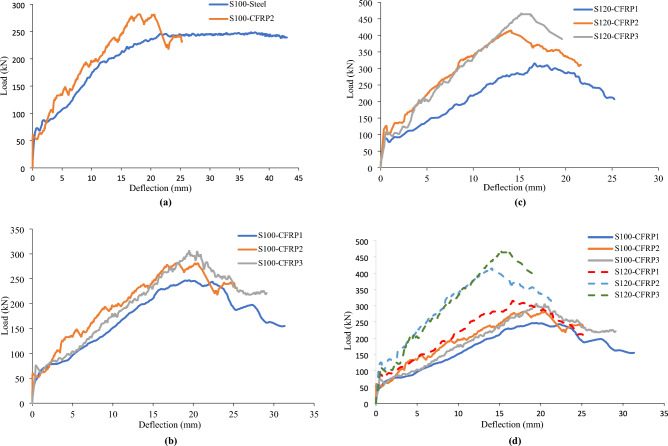
Experimental load–deflection curves of the tested slabs: (**a**) comparison between the steel-reinforced control specimen S100-Steel and the CFRP-reinforced specimen S100-CFRP2; (**b**) Group A specimens with constant slab thickness of 100 mm and different CFRP reinforcement ratios; (**c**) Group B specimens with constant slab thickness of 120 mm and different CFRP reinforcement ratios; (**d**) comparison between Group A and Group B specimens.

Overall, the results demonstrate that replacing steel reinforcement with CFRP bars significantly reduces ultimate deflection, while increasing slab thickness further improves stiffness and deformation control, as illustrated in Fig. 5 through the load–deflection curves.

### Crack pattern and mode of failure

**Fig. 6:**
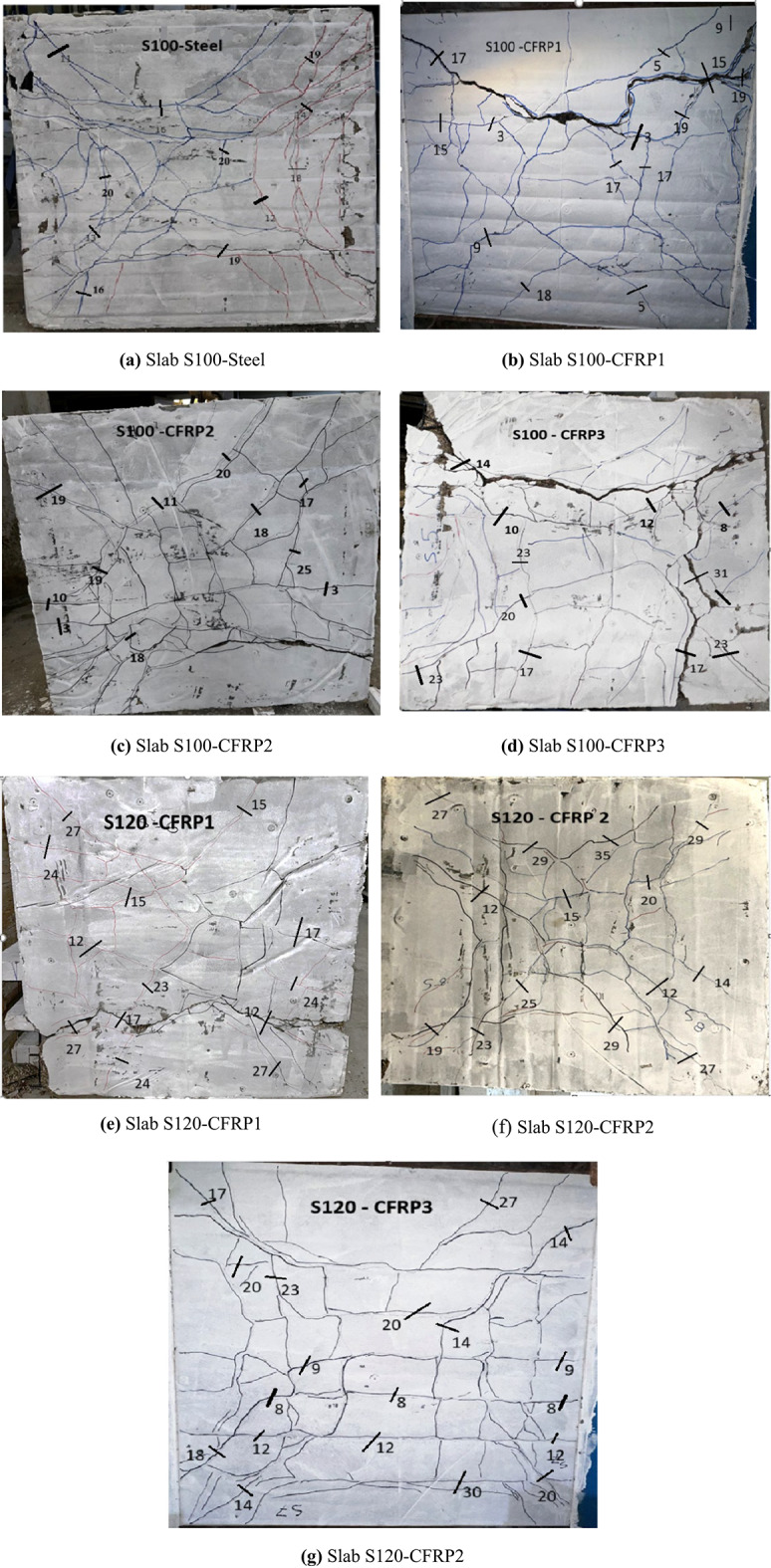
Crack patterns of the tested slabs. The numerical values shown on the crack maps indicate the applied load level in Ton at which the corresponding cracks were first observed and marked during testing.

The crack propagation and failure modes of the two-way concrete slabs reinforced with either CFRP or steel bars were observed and documented throughout the loading process until failure. Crack development on the tension face (bottom surface) was carefully monitored and marked at each loading stage. Figure 6 presents the crack patterns of all tested slabs, while Table 4 summarizes the corresponding failure modes. The numerical values shown on the crack maps represent the applied load levels in Ton at which the cracks were first observed during testing. For the control slab (S100–Steel), cracks initiated in the tension zone and propagated in a well-distributed pattern across the slab, indicating a typical ductile flexural behavior.

**Table 4:** Observed failure modes of the tested slabs.

Slab ID	Failure mode	Description
S100-Steel	DF	Ductile flexural failure with distributed cracking
S100-CFRP1	BF	Brittle flexural failure with dominant crack formation
S100-CFRP2	BF	Flexural failure with relatively localized cracking
S100-CFRP3	BF	Brittle flexural failure with major crack propagation
S120-CFRP1	BF	Brittle flexural failure with localized crack development
S120-CFRP2	BF	Flexural failure with moderate crack distribution
S120-CFRP3	BF	Flexural failure with relatively uniform crack pattern

For CFRP-reinforced slabs in the S100 series, crack propagation was characterized by the formation of dominant cracks with fewer secondary cracks, reflecting a more localized cracking pattern. This behavior indicates a relatively brittle flexural response compared to the steel-reinforced slab. A similar trend was observed for the **S120 series**; however, cracks appeared less dense and more confined, suggesting improved structural performance with increased slab thickness.

Overall, all slabs exhibited flexural failure as the governing mode. A clear distinction was observed between the distributed cracking and ductile behavior of the steel-reinforced slab and the more localized, relatively brittle response of the CFRP-reinforced slabs.

### Toughness

Flexural toughness was calculated as the area under the experimental load–deflection curve from zero load to the deflection corresponding to the ultimate load point, as given in Eq. (3):

3 $$Toughness={\int}_{0}^{{\Delta}_{u}}P\left(\Delta \right)\hspace{0.17em}d\Delta$$

where *P* is the applied load,* Δ* is the measured mid-span deflection, and *Δ*_*u*_ is the deflection corresponding to the ultimate load point.

Therefore, the reported toughness values represent the energy absorption capacity of the tested slabs up to the peak load.

The results show that the steel-reinforced slab exhibited the highest toughness value of 8815 kN·mm. The toughness values of the CFRP-reinforced slabs were lower than that of the steel-reinforced slab by approximately 35–43% for the 100 mm slabs and 27–38% for the 120 mm slabs, as shown in Table 5**.**

**Table 5:** Experimental results of tested slabs.

Group	Slab ID	P_u_​ (kN)	Δ_u_ (mm)	P_cr_ (kN)	Deformability Index	Toughness (kN·mm)	*K*_*sec*_ ​(kN/mm)
**Control**	S100-Steel	249.10	36.91	80	30.8	8815	16.6
**Group** **A**	S100-CFRP1	247.43	19.47	70	19.5	5357	15.9
	S100-CFRP2	281.65	18.16	90	12.1	5011	21.7
	S100-CFRP3	307.46	19.49	85	16.2	5724	19.2
**Group** **B**	S120-CFRP1	316.14	16.64	75	16.6	5480	26.3
	S120-CFRP2	414.37	14.13	110	7.1	6447	41.4
	S120-CFRP3	467.73	15.20	100	8.4	5988	39.0

Note: P_u_ is the ultimate load, *Δ*_*u*_ is the deflection at ultimate load, P_cr_ is the first cracking load, and *K*_*sec*_ is the secant stiffness evaluated at 50% of the ultimate load. Toughness was calculated as the area under the experimental load–deflection curve from zero load up to the ultimate load point.

For the CFRP-reinforced slabs, the effect of reinforcement ratio on toughness was not strictly monotonic. In the 100 mm slabs, S100-CFRP3 showed approximately 6.9% higher toughness than S100-CFRP1, whereas S100-CFRP2 showed a lower toughness value due to its lower deformation capacity. In the 120 mm slabs, toughness increased by approximately 17.6% for S120-CFRP2 and 9.3% for S120-CFRP3 relative to S120-CFRP1. In addition, increasing slab thickness from 100 to 120 mm improved toughness by approximately 2–29%.

Overall, despite the higher load capacity of some CFRP-reinforced slabs, their toughness was reduced by up to 43% compared with the steel-reinforced slab due to their lower deformation capacity and the linear-elastic brittle behavior of CFRP reinforcement.

### Deformability index

The deformability index was defined as the ratio between the ultimate deflection and the deflection at first cracking. In this study, the ultimate deflection was defined as the measured mid-span deflection corresponding to the ultimate load point on the experimental load–deflection curve, while the cracking deflection was taken at the first cracking load. This index was used to compare the deformation capacity of the tested slabs because CFRP reinforcement behaves linearly up to rupture without yielding. The deformability index was calculated using Eq. (4):

4 $$DI=\frac{{\Delta}_{u}}{{\Delta}_{cr}}$$

where *DI* is the deformability index, *Δ*_*u*_ is the deflection at ultimate load, and *Δ*_*cr*_ is the deflection at first cracking load.

The results indicate that the steel-reinforced slab exhibited the highest deformability index of 30.8, while the CFRP-reinforced slabs showed significantly lower values ranging from 7.1 to 19.5, representing a reduction of approximately 37–77%, as shown in Table 5**.** This reduction is mainly attributed to the linear-elastic behavior and brittle failure characteristics of CFRP reinforcement. Additionally, increasing slab thickness resulted in lower deformability due to the increase in stiffness, while the effect of reinforcement ratio on deformability was found to be inconsistent.

### Secant stiffness

The secant stiffness at 50% of the ultimate load was evaluated for all tested slabs, as presented in Table 5. Under identical slab thickness (100 mm) and nominal reinforcement ratio (0.42%), the CFRP-reinforced slab S100-CFRP2 exhibited approximately 30% higher global secant stiffness (21.7 kN/mm) than the steel-reinforced reference slab S100-Steel (16.6 kN/mm).

This global secant stiffness should be distinguished from the effective reinforcement stiffness, ${{\boldsymbol{\rho}}}_{{\boldsymbol{h}},{\boldsymbol{s}}}$, reported in Table 3. Although S100-CFRP2 had lower effective reinforcement stiffness than S100-Steel due to the lower elastic modulus of CFRP bars, it showed higher measured global secant stiffness. This apparent contradiction is mainly because *K*_*sec*_ represents the overall load–deflection response of the slab system at 50% of the ultimate load, not the elastic stiffness of the reinforcement material alone.

Since S100-CFRP2 reached a higher ultimate load than S100-Steel, the secant stiffness was evaluated at a higher load level. In addition, the ribbed surface of the CFRP bars may have improved mechanical interlock with the surrounding concrete and reduced reinforcement slip after cracking. In contrast, the steel-reinforced slab developed larger deformation capacity due to yielding and stress redistribution before failure. Therefore, the reported secant stiffness should not be used as a direct comparison between the elastic moduli of steel and CFRP reinforcement.

When comparing specimens with different thicknesses, the 120 mm CFRP-reinforced slabs showed global secant stiffness increases of approximately 58–149% relative to the 100 mm steel-reinforced slab. These larger increases reflect the combined effects of greater slab thickness, reinforcement ratio, and reinforcement system, rather than the CFRP material alone. Overall, stiffness increased with slab thickness and reinforcement ratio, as expected from flexural theory.

This apparent stiffness issue is further discussed mechanistically in Section "Clarification of the apparent stiffness contradiction".

### Discussion of observed mechanisms

To clarify the mechanistic contribution of the study, the observed response is interpreted through the interaction between reinforcement stiffness, strength-based mechanical reinforcement index, post-cracking stress redistribution, slab thickness, crack localization, deformability, toughness, and global secant stiffness. This interpretation explains the governing mechanisms behind the experimental trends rather than merely reporting ultimate load values.

#### Mechanically normalized comparison between steel- and CFRP-reinforced slabs

Although S100-Steel and S100-CFRP2 had the same nominal reinforcement ratio of 0.42% and the same slab thickness of 100 mm, they were not mechanically equivalent because steel and CFRP reinforcement have different elastic moduli, tensile strengths, and stress–strain behavior. Therefore, the comparison was interpreted using the mechanical reinforcement index, ${\rho}_{h,f}$, and the effective reinforcement stiffness, ${\rho}_{h,s}$, rather than the nominal reinforcement ratio alone.

The results show that S100-CFRP2 had lower effective reinforcement stiffness than S100-Steel, reflecting the lower elastic modulus of CFRP reinforcement. However, S100-CFRP2 had a higher mechanical reinforcement index due to the high tensile strength of CFRP bars. This explains why S100-CFRP2 achieved an approximately 13% higher ultimate load than S100-Steel, despite not having higher reinforcement stiffness. Accordingly, the higher ultimate load should be interpreted as a strength-based and slab-system response governed by CFRP tensile capacity, post-cracking behavior, and internal force redistribution, rather than as a direct material superiority of CFRP over steel at the same nominal reinforcement ratio.

#### Lower deformation capacity of CFRP-reinforced slabs

The steel-reinforced slab exhibited 36.91 mm ultimate deflection compared to 18.16 mm for the CFRP slab with identical reinforcement ratio and thickness (S100-CFRP2). This 50.8% reduction is explained by the linear-elastic behavior of CFRP up to rupture (no yielding) and its lower elastic modulus (150 GPa for CFRP vs. 200 GPa for steel). The absence of yielding eliminates the post-yield deformation reserve that characterizes steel-reinforced flexural members. Additionally, the higher bond strength and stiffness of CFRP bars (ribbed surface) limit slip and concentrated curvature development, further reducing deflection capacity.

#### Localized cracking in CFRP-reinforced slabs

CFRP-reinforced slabs developed fewer but wider cracks compared to the steel-reinforced slab. This is attributed to the lower elastic modulus and linear-elastic rupture of CFRP, which limits stress redistribution after cracking. In steel-reinforced concrete, yielding allows redistribution of tensile stresses across multiple cracks, promoting distributed cracking. In contrast, CFRP bars maintain elastic stress concentration at the first major crack until rupture, resulting in localized failure with one or two dominant cracks.

#### Effect of slab thickness on capacity and stiffness

Increasing slab thickness from 100 to 120 mm enhanced ultimate load by 27.7–52.1% and secant stiffness by 26–105% (depending on reinforcement ratio). This follows directly from flexural theory: cracking moment is proportional to thickness squared (${M}_{cr}\propto {h}^{2})$, and ultimate moment capacity increases with effective depth ( ${M}_{n}\propto d$). The thicker section also provides a larger lever arm between tensile and compressive forces, reducing cracking intensity and delaying failure.

#### Effect of reinforcement ratio on CFRP slabs

Within each thickness group, increasing the CFRP reinforcement ratio from 0.35% to 0.50% increased ultimate load by 13.8–24.3% (100 mm slabs) and 31.1–48.0% (120 mm slabs). The more pronounced effect in thicker slabs is due to the larger effective depth, which allows higher CFRP tensile forces to be fully utilized before concrete crushing. The non-proportional increase (i.e., 48% capacity increase for 43% area increase) reflects the improved strain compatibility and delayed neutral axis rise in thicker, more heavily reinforced sections.

#### Reduction in toughness and deformability

The steel-reinforced slab exhibited 30–43% higher toughness than CFRP-reinforced slabs despite lower ultimate load. This apparent paradox is resolved by recognizing that toughness is the area under the load–deflection curve. Steel reinforcement yields and sustains load over large deflections, producing a long tail in the curve. CFRP reinforcement ruptures suddenly after linear-elastic response, truncating the curve. Similarly, the deformability index (ultimate deflection / cracking deflection) dropped from 30.8 (steel) to 7.1–19.5 (CFRP), reflecting the absence of yielding and the abrupt failure characteristic of FRP materials.

These mechanisms are consistent with classical flexural theory and with previous observations in FRP-reinforced concrete beams and one-way slabs, but are quantified here specifically for two-way HSC slabs reinforced with CFRP bars.

#### Clarification of the apparent stiffness contradiction

A notable observation is that S100-CFRP2 exhibited higher global secant stiffness than S100-Steel, although CFRP bars have a lower elastic modulus than steel reinforcement. This apparent contradiction can be explained by distinguishing between reinforcement material stiffness and slab-system secant stiffness. The reported secant stiffness was evaluated at 50% of the ultimate load from the global load–deflection response. Since S100-CFRP2 reached a higher ultimate load than S100-Steel, the stiffness was evaluated at a higher load level for the CFRP-reinforced slab.

In addition, CFRP reinforcement behaves linearly up to rupture without yielding, whereas the steel-reinforced slab developed larger deformation capacity due to yielding and stress redistribution before failure. The more localized cracking observed in the CFRP-reinforced slab may also have allowed larger uncracked regions of the slab to contribute to the global response. Furthermore, the ribbed surface of the CFRP bars may have improved mechanical interlock with the surrounding concrete and reduced reinforcement slip after cracking.

Therefore, the higher global secant stiffness of S100-CFRP2 is a system-level response and does not contradict the lower elastic modulus of CFRP bars. This result should not be interpreted as evidence that CFRP reinforcement is stiffer than steel reinforcement at the material level.

## Nonlinear finite element analysis

Nonlinear finite element analysis (NLFEA) was conducted using ABAQUS/CAE 2020^41^ to simulate the response of high-strength concrete slabs reinforced with steel and CFRP bars under uniformly distributed loading. The finite element models were established to reproduce the experimental configuration and to evaluate the key structural responses, including load–deflection behavior, crack development, and ultimate load-carrying capacity.

### Finite element modelling

#### Element types

**Fig. 7:**
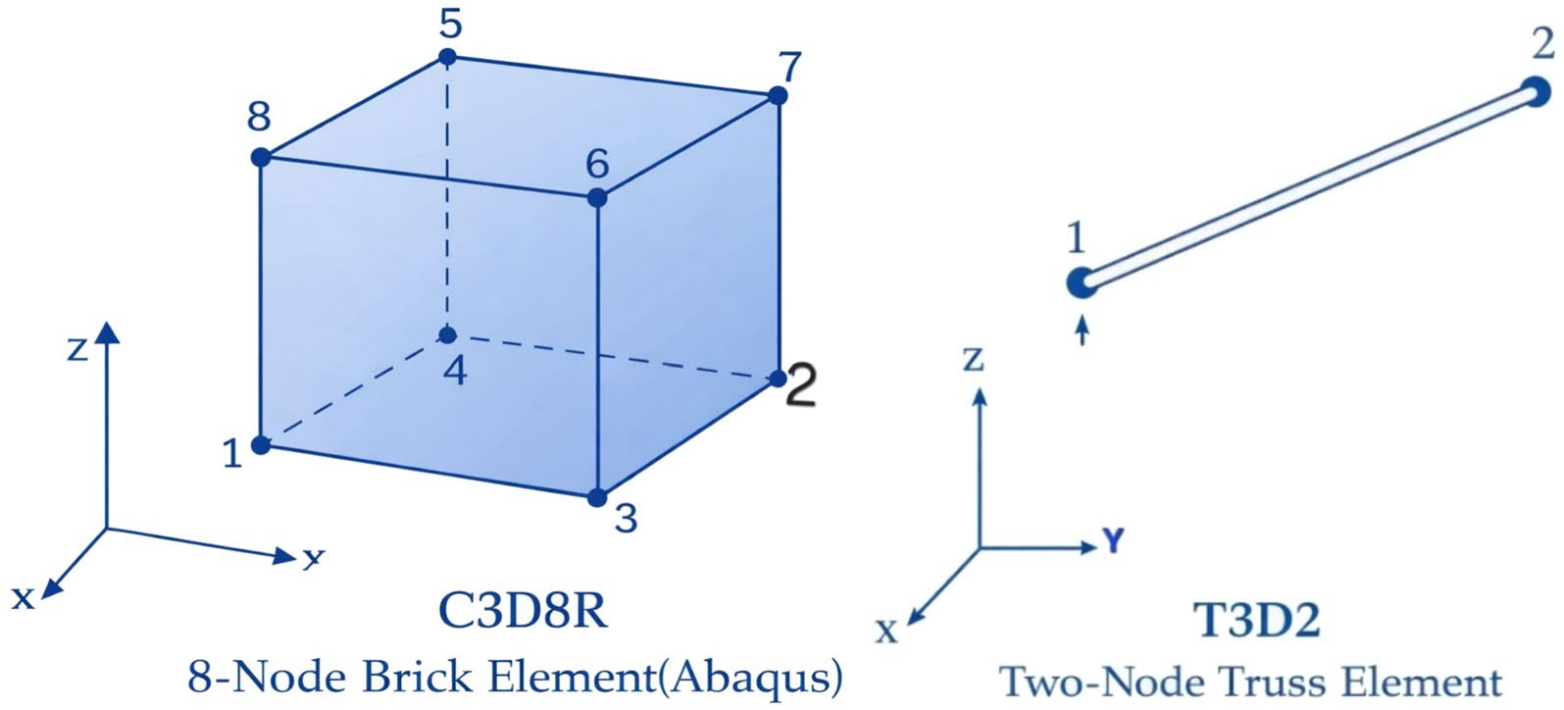
(**a**-**b**) T3D2 truss elements and C3D8R solid elements adopted for modelling the reinforcement and concrete, respectively.

The concrete slab was modeled using three-dimensional eight-node reduced-integration solid elements (C3D8R), whereas the steel and CFRP reinforcement bars were represented using two-node truss elements (T3D2), as shown in Fig. 7**.** These element types were selected because they provide an efficient and reliable representation of the nonlinear response of reinforced concrete slabs under flexural loading.

#### Finite element model setup

**Fig. 8:**
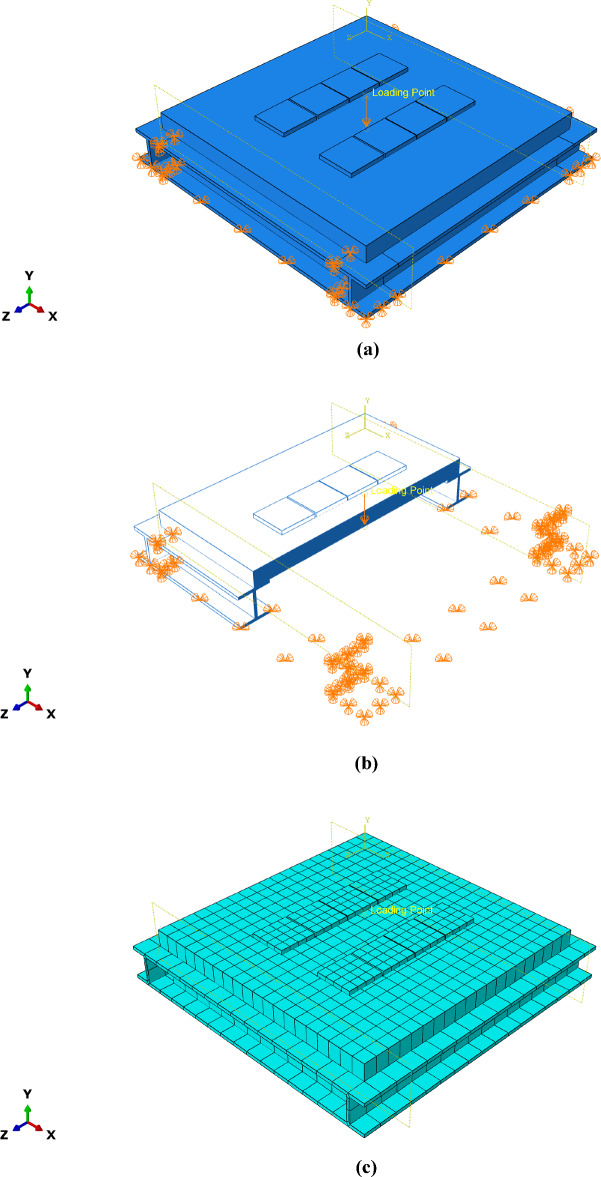
Finite element model of the HSC slab: (**a**) overall model geometry; (**b**) boundary conditions and loading configuration; (**c**) mesh discretization.

The finite element model was constructed to simulate the geometry, boundary conditions, and loading scheme of the tested HSC slabs. The concrete slab and reinforcement layout were represented in three dimensions to ensure consistency with the experimental setup. Simply supported conditions were assigned along the slab edges, while the external load was applied as a uniformly distributed load to reproduce the experimental loading arrangement, as illustrated in Fig. 8**.**

A uniform mesh size of 75 mm was adopted to achieve an appropriate balance between computational efficiency and numerical accuracy. This mesh size provided stable numerical convergence while maintaining a suitable representation of the load–deflection response and damage distribution. A preliminary mesh sensitivity study was conducted; reducing the mesh size from 75 to 50 mm changed the predicted ultimate load by less than 3%, confirming that the chosen mesh size provides mesh‑independent results.

The interaction between the concrete and reinforcement was defined using the Embedded Region constraint available in ABAQUS, in which the reinforcement elements were embedded within the concrete host elements. This modeling approach assumes a perfect bond condition between the concrete and reinforcement, ensuring full strain compatibility and eliminating relative slip throughout the loading process. Although bond-slip behavior was not explicitly modeled, this assumption was considered acceptable in the present study because no premature bond failure was observed experimentally, and the numerical model was mainly intended to capture the global structural response, including ultimate load, load–deflection behavior, and damage distribution.

### Material properties

**Concrete: based on ECP 203/2018**^37^—For *f*_*cu*_ = 75 MPa, *E*_*c*_ was taken as approximately 39.8 GPa.—Poisson’s ratio: *ν* = 0.20—CDP parameters: dilation angle = 30°, eccentricity = 0.1, *f*_*bo*_*/f*_*co*_ = 1.16, *K* = 0.67, viscosity = 0.0005.

**Steel reinforcement: based on ECP 203/2018 **^37^—Elastic modulus: *E*_*s*_ = 200 GPa—Yield stress: *f*_*y*_ = 420 MPa—Poisson’s ratio: *ν* = 0.30—Modeled as elastic–plastic with isotropic hardening.

CFRP reinforcement: Young’s modulus *E*_*f*_ = 150 GPa, tensile strength *f*_*fu*_ = 1400 MPa, and Poisson’s ratio: *ν* = 0.30. The CFRP bars were modeled as linear elastic up to rupture, reflecting their brittle tensile behavior.

*Where: Ec* = modulus of elasticity of concrete, *ν* = Poisson’s ratio, $f_{u}$ = concrete cube compressive strength, *f*_*y*_ = yield stress of steel reinforcement, $E_f$ = elastic modulus of steel reinforcement, *E*_*f*_ = elastic modulus of CFRP reinforcement, *f*_*fu*_ = ultimate tensile strength of CFRP reinforcement.

### NLFEA results and discussion

#### NLFEA ultimate load and deflection

**Fig. 9:**
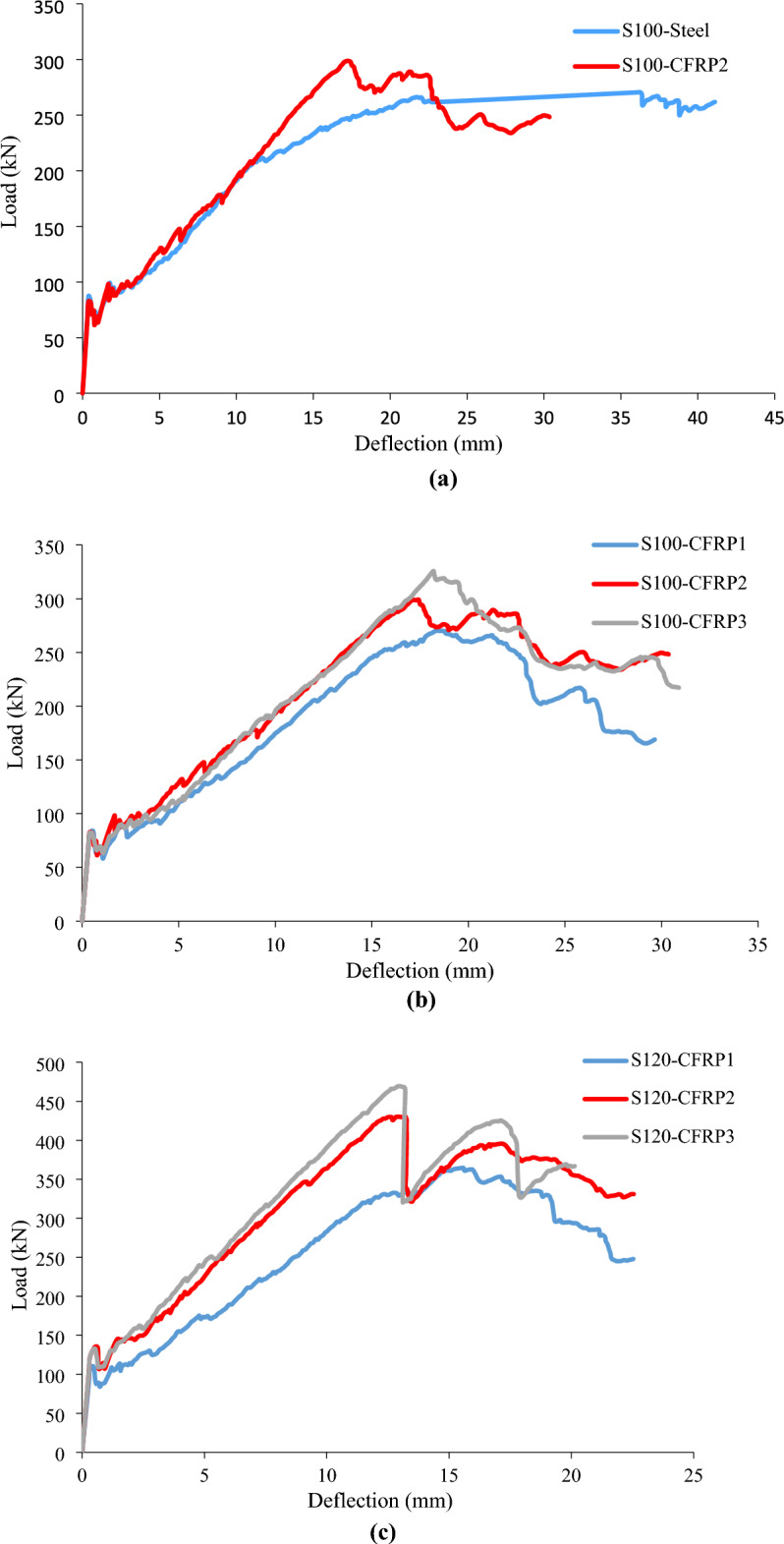
Numerical load–deflection curves obtained from the finite element models: (**a**) comparison between the steel-reinforced control specimen S100-Steel and the CFRP-reinforced specimen S100-CFRP2; (**b**) Group A specimens with constant slab thickness of 100 mm and different CFRP reinforcement ratios; (**c**) Group B specimens with constant slab thickness of 120 mm and different CFRP reinforcement ratios.

As shown in Fig. 9 and Table 6 for the 100 mm slabs, the steel control specimen (S100–Steel) reached an ultimate load of 271.14 kN with a corresponding deflection of 36.35 mm, exhibiting higher deformation capacity compared to CFRP-reinforced slabs.

**Table 6:** Numerical results of tested slabs.

Group	ID	P_u*, Num*_ (kN)	*Δ*_*u, Num*_ (mm)	P_cr*, Num*_(kN)
Control	S100-Steel	271.14	36.3548	85
Group A	S100-CFRP1	270.79	18.4078	84
	S100-CFRP2	299.216	17.4131	92
	S100-CFRP3	325.82	16.1954	86.4
Group B	S120-CFRP1	364.45	15.592	109.75
	S120-CFRP2	431.18	12.61	133.26
	S120-CFRP3	469.69	11.9528	132.65

Note: P_u*, Num*_ is the numerical ultimate load, *Δ*_*u, Num*_ is the deflection at numerical ultimate load, and.

The CFRP specimens (S100–CFRP1, S100–CFRP2, and S100–CFRP3) achieved ultimate loads of 270.79 kN, 299.22 kN, and 325.82 kN, respectively, with corresponding deflections of 18.41 mm, 17.41 mm, and 16.20 mm. This indicates an increase in load capacity with increasing reinforcement ratio, accompanied by a reduction in deformation capacity.

For the 120 mm slabs, increasing slab thickness resulted in a significant enhancement in both stiffness and ultimate load. Specimens S120-CFRP1, S120-CFRP2, and S120-CFRP3 reached ultimate loads of 364.45 kN, 431.18 kN, and 469.69 kN, respectively, with corresponding deflections of 15.59 mm, 12.61 mm, and 11.95 mm.

Among all specimens, S120-CFRP3 exhibited the highest load-carrying capacity (469.69 kN) and the lowest deformation, highlighting the combined effect of increased slab thickness and reinforcement ratio.

Overall, the numerical results confirm that increasing reinforcement ratio and slab thickness leads to enhanced structural performance in terms of strength and stiffness, while reducing deformation capacity.

P_cr, *Num*_ is the numerical first cracking load obtained from the finite element model.

#### Numerical damage distribution and failure mechanism

**Fig. 10:**
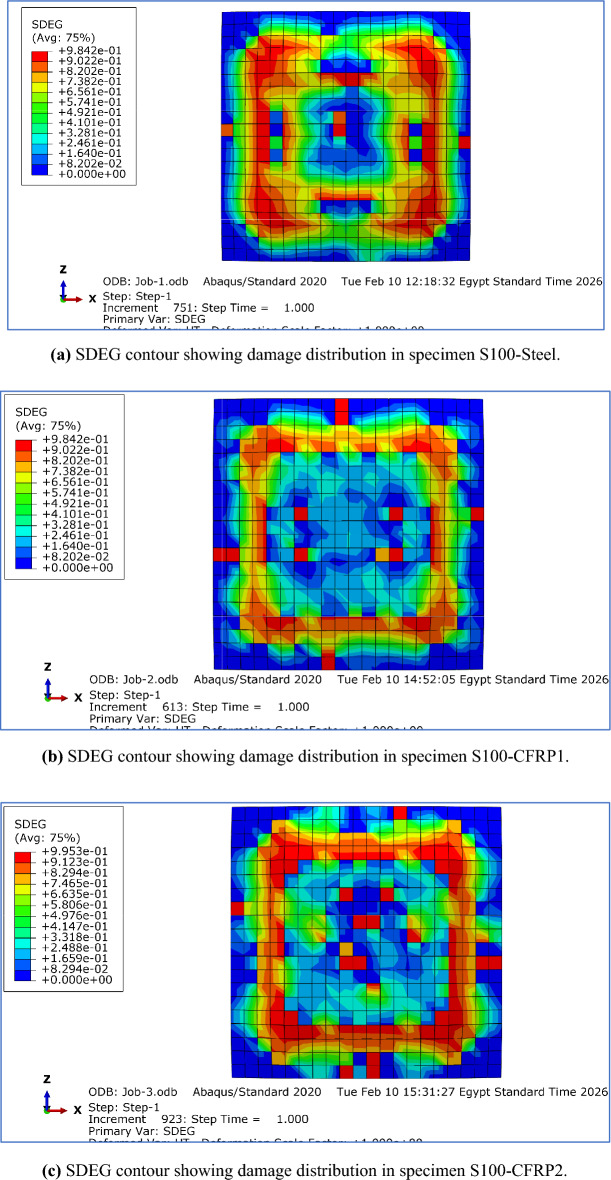

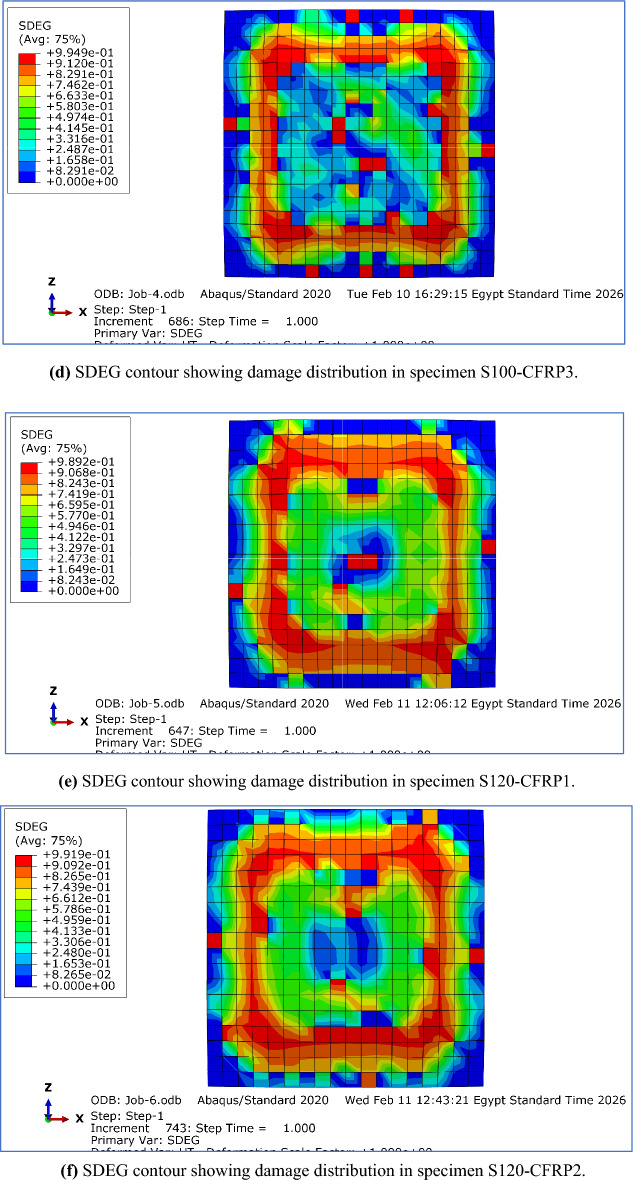

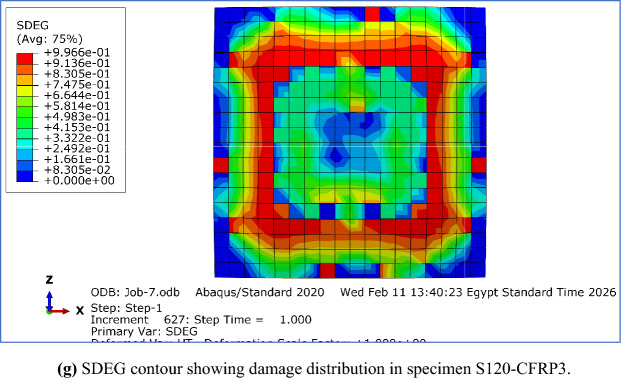
Distribution of the scalar stiffness degradation parameter (SDEG) obtained from the ABAQUS numerical model, illustrating the development and propagation of concrete damage.

In ABAQUS, the scalar stiffness degradation parameter (SDEG) is used to represent the damage state of concrete. The value of SDEG ranges from 0 to 1, where 0 indicates undamaged concrete, while a value approaching 1 represents complete stiffness degradation and severe material damage. Therefore, the SDEG contours shown in Fig. 10 were used to visualize the initiation and propagation of concrete damage in the numerical models.

Damage initiated in the tension zone and localized toward the slab center at advanced loading stages, defining the governing failure regions. Increasing slab thickness reduced damage intensity and promoted a more confined damage response. In contrast, the CFRP-reinforced slabs exhibited more pronounced damage localization due to the limited stress redistribution associated with the linear-elastic behavior of CFRP bars, indicating a relatively brittle failure mechanism. Overall, the numerical results provide additional insight into damage progression beyond the experimental observations.

### Comparisons between Experimental and NLFEA Results

The developed finite element model was validated by comparing the numerical results with the experimental data in terms of load–deflection response, ultimate load, ultimate deflection, and first crack load.

#### Comparison between experimental and NLFEA ultimate loads

**Fig. 11:**
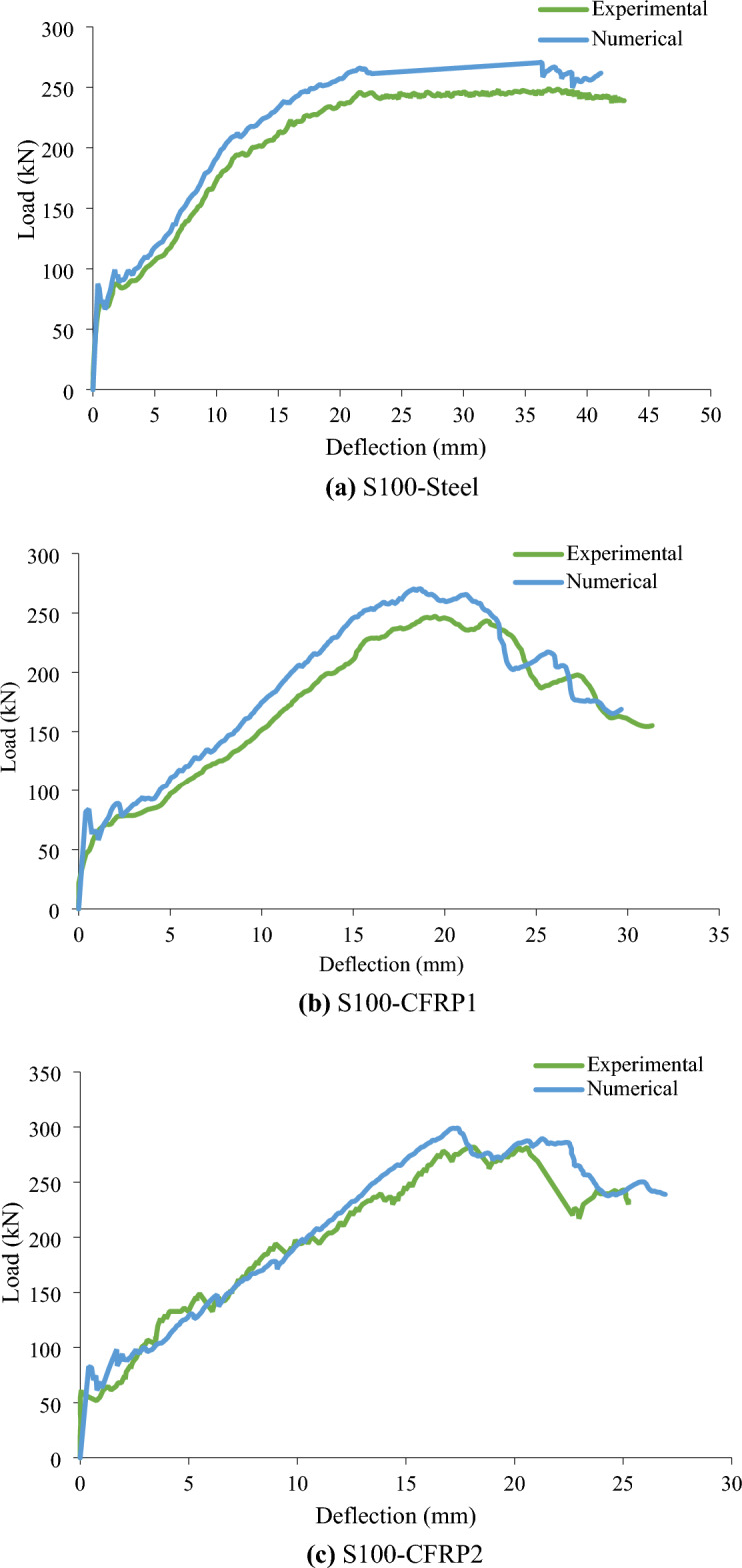

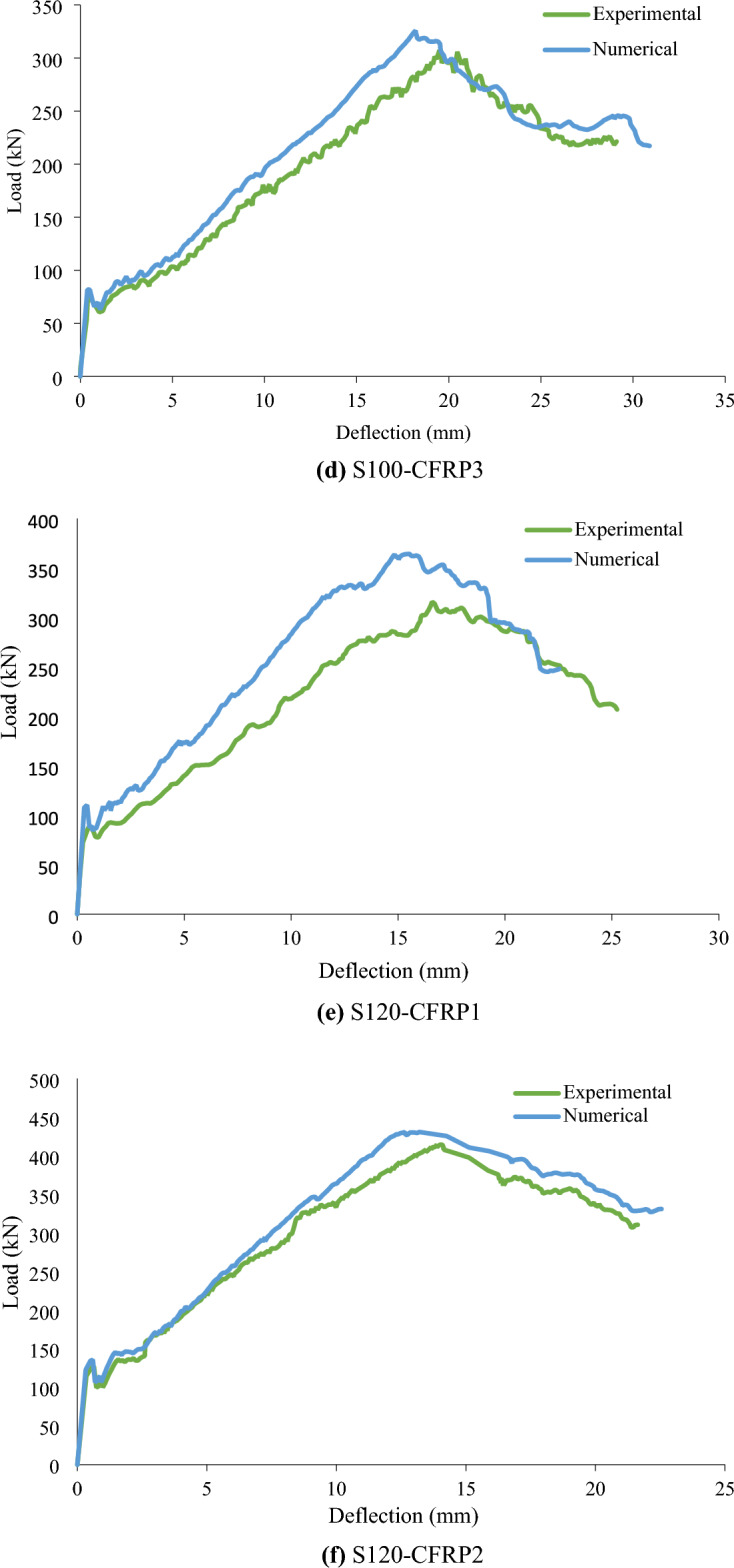

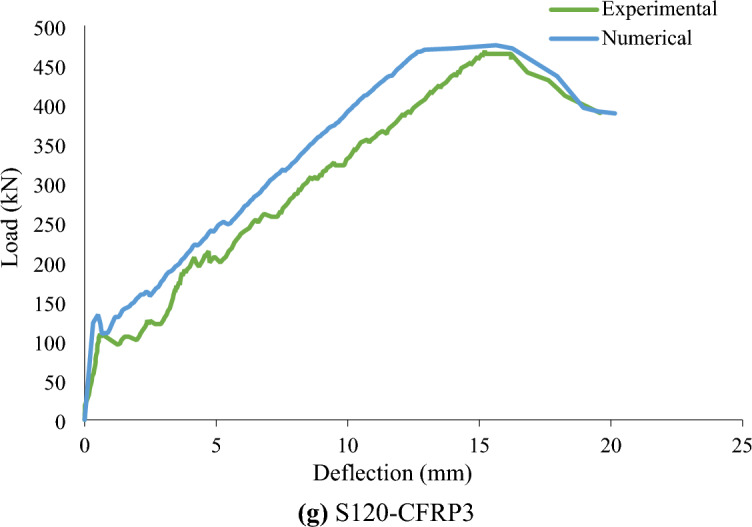
Comparison between experimental and numerical load–deflection responses of all tested slabs: (**a**) S100-Steel; (**b**) S100-CFRP1; (**c**) S100-CFRP2; (**d**) S100-CFRP3; (**e**) S120-CFRP1; (**f**) S120-CFRP2; and (**g**) S120-CFRP3.

Figure 11 shows good agreement between numerical and experimental results for all tested slabs. The ratio of experimental to numerical ultimate load ranged from 0.87 to 1.00, with an average value of 0.93 and a standard deviation of 0.04 as found in Table 6**.**

**Fig. 12:**
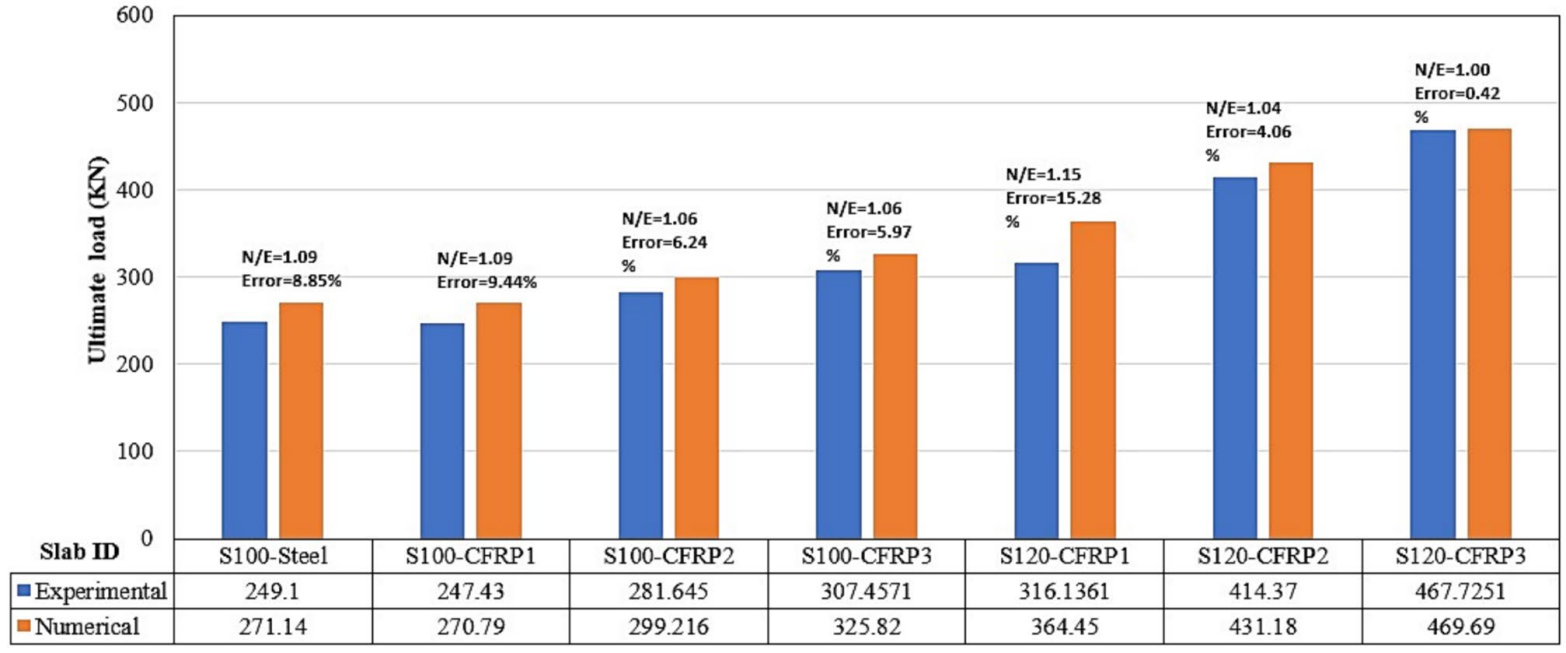
Comparison between experimental and numerical ultimate load values of tested slabs.

The numerical model generally predicted slightly higher ultimate load values than the experimental results. For example, the ultimate load of specimen S100-Steel increased from 249.10 kN experimentally to 271.14 kN numerically, while for S120-CFRP3, the values were very close (467.73 kN experimentally and 469.69 kN numerically). The prediction accuracy was evaluated using the numerical-to-experimental ratio (N/E) and the corresponding percentage error for each slab specimen, as shown in Fig. 12.

#### Comparison between experimental and NLFEA deflections

**Fig. 13:**
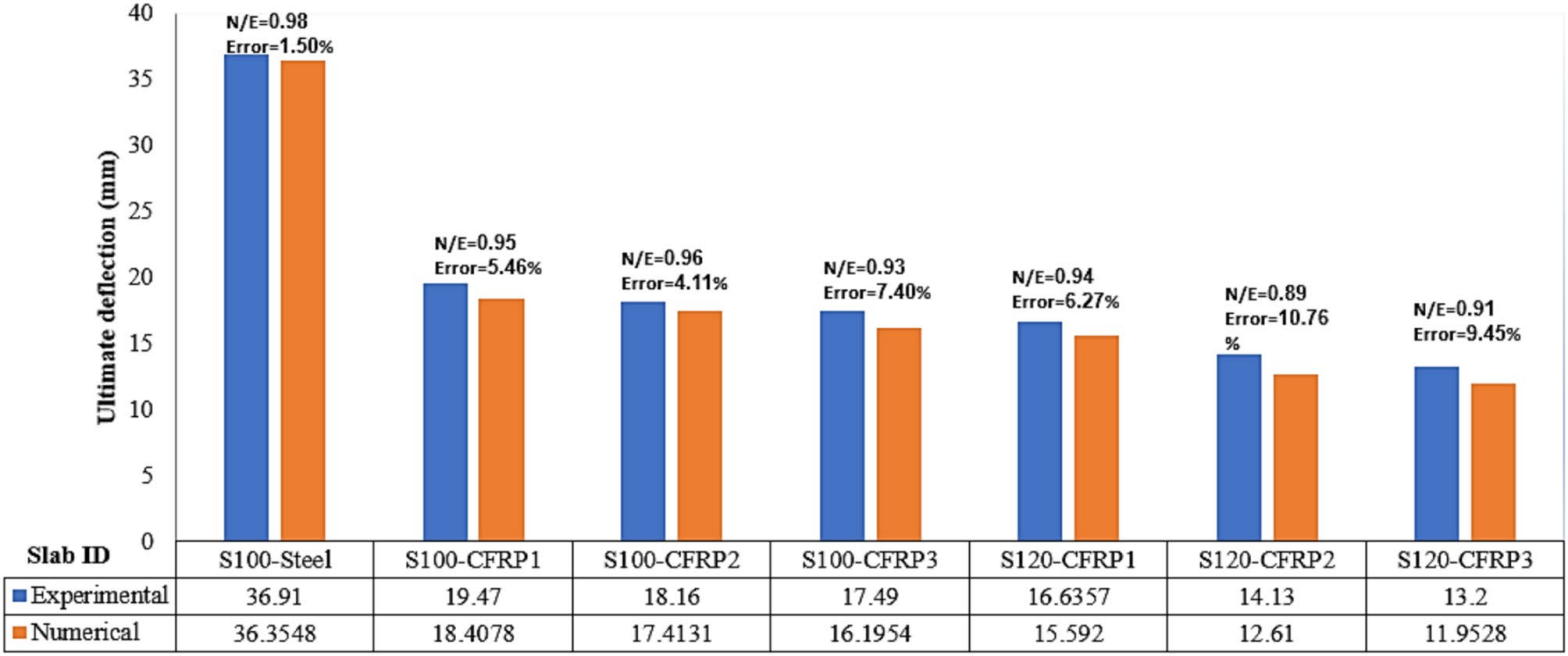
Comparison between experimental and numerical ultimate deflection values of tested slabs.

The comparison of ultimate deflection as illustrated in Fig. 13 values indicates that the numerical model slightly underestimates the deformation capacity of the slabs. Table 7 shows that the ratio of experimental to numerical deflection ranged from 1.02 to 1.12, with an average value of 1.07 and a standard deviation of 0.03.

**Table 7:** Comparison between experimental and numerical results.

**Slab ID**	**Experimental**		**Numerical**		**P**_***u,Exp***_*** /*** **P**_***u,Num***_	**Δ **_***Exp.***_*** /*** **Δ **_***Num***_
	**P**_**u*,Exp***_ **(kN)**	**Δ**_***Exp***_ **(mm)**	**P**_***u,Num.***_** (kN)**	***Δ***_***Num.***_** (mm)**		
S100-Steel	249.10	36.91	271.14	36.35	0.92	1.02
S100-CFRP1	247.43	19.47	270.79	18.41	0.91	1.06
S100-CFRP2	281.65	18.16	299.22	17.41	0.94	1.04
S100-CFRP3	307.46	17.49	325.82	16.20	0.94	1.08
S120-CFRP1	316.14	16.64	364.45	15.59	0.87	1.07
S120-CFRP2	414.37	14.13	431.18	12.61	0.96	1.12
S120-CFRP3	467.73	13.20	469.69	11.95	1.00	1.10
Average	-	-	-	-	0.93	1.07
Standard deviation	-	-	-	-	0.04	0.03

Note: P_*u,Exp.*_ is the experimental ultimate load, P_*u,Num.*_ is the numerical ultimate load obtained from the finite element model*,* Δ_*u,Exp.*_ is the experimental deflection at ultimate load, and Δ_*u,Num.*_ is the numerical deflection at ultimate load. The ratios P_*u,Exp*_*./*P_*u,Num.*_ and Δ_*u,Exp*_*./*Δ_*u,Num*_. represent the experimental-to-numerical ratios.

For instance, the ultimate deflection of specimen S100–Steel decreased slightly from 36.91 mm experimentally to 36.35 mm numerically. Similarly, for S120-CFRP2, the deflection decreased from 14.13 mm to 12.61 mm.

#### Comparison between Experimental and NLFEA first crack and crack pattern

**Fig. 14:**
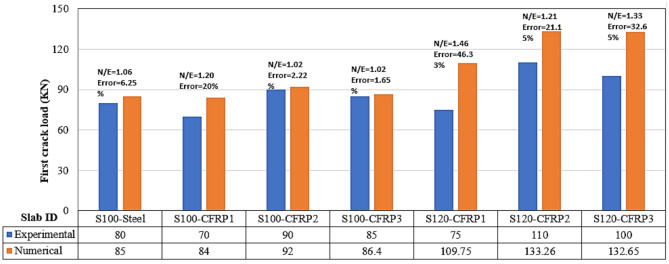
Comparison between experimental and numerical first crack load for all tested slabs.

**Fig. 15:**
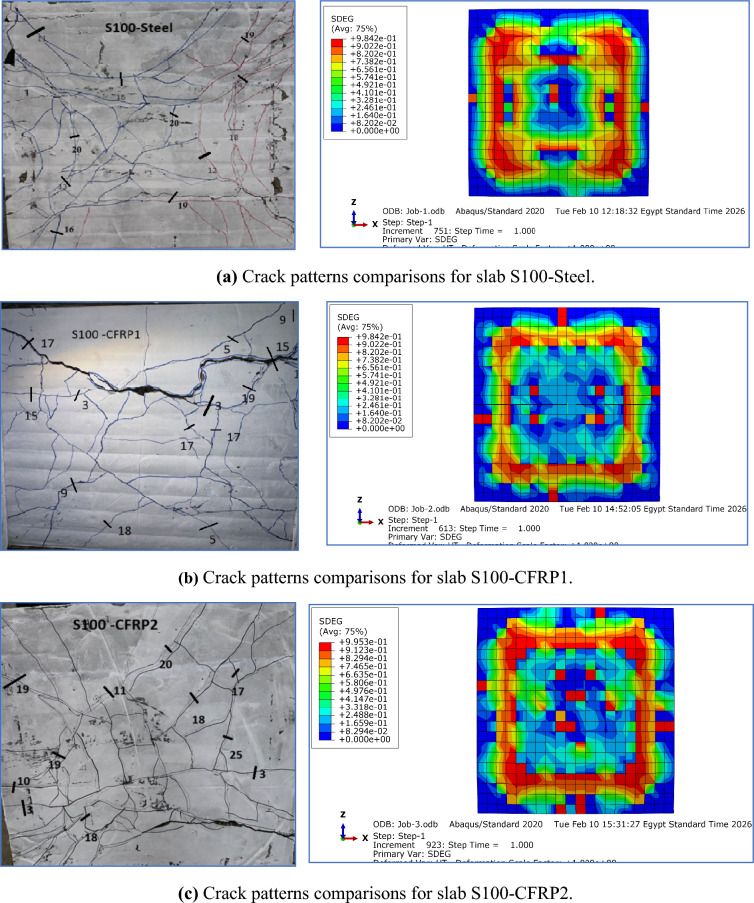

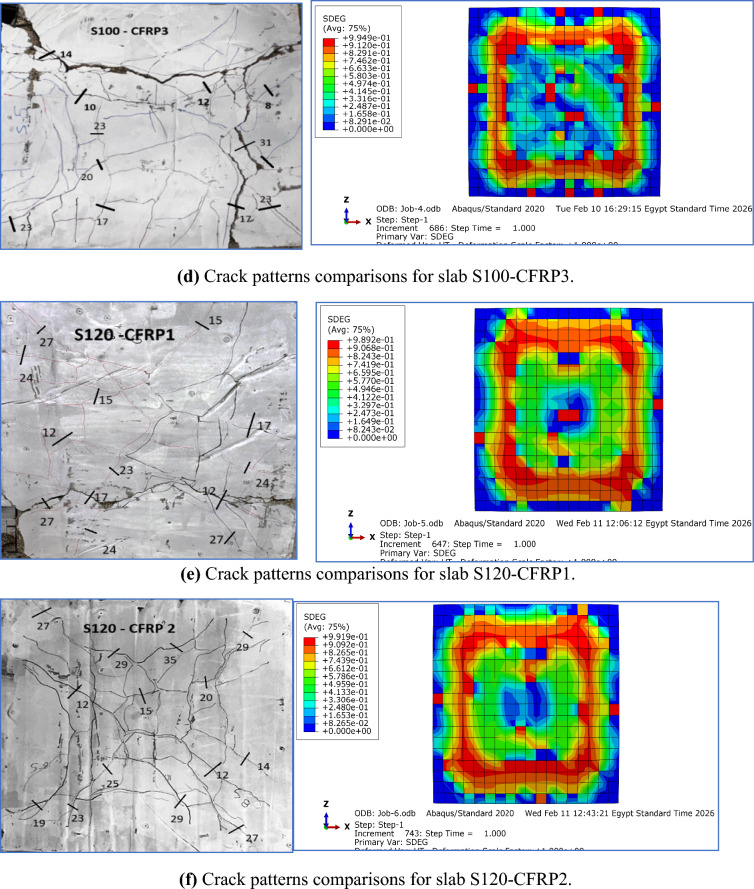

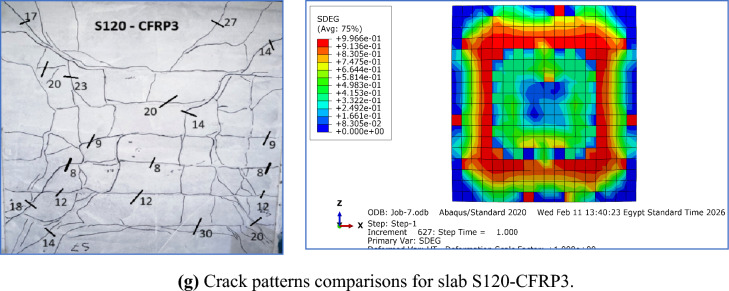
Crack patterns for all specimens.

The comparison of first crack load values shows generally good agreement between the experimental and numerical results as shown in Fig. 14**.** The crack patterns obtained from the finite element analysis showed good agreement with the experimental observations in terms of crack distribution and propagation as observed in Fig. 15. In both experimental and numerical results, cracks were primarily initiated at the tension zone near the bottom surface and propagated towards the slab center, forming typical two-way flexural crack patterns under uniformly distributed loading.

In the experimental specimens, cracks appeared more irregular and widely distributed, with the presence of multiple fine cracks and, in some cases, dominant major cracks indicating localized stress concentrations. In contrast, the numerical results exhibited a more uniform and symmetric crack distribution due to the idealized modeling assumptions and the homogeneous material representation in the finite element model. Nevertheless, the numerical model successfully captured the overall cracking behavior and the critical regions of stress concentration.

Regarding the failure mode, all tested slabs exhibited flexural failure behavior. The steel-reinforced slab demonstrated a ductile flexural response characterized by gradual crack development and distributed cracking over a larger area. On the other hand, CFRP-reinforced slabs generally showed a more brittle flexural failure, marked by the formation of dominant cracks and relatively sudden propagation at ultimate load. Although some CFRP specimens exhibited relatively more distributed cracking, their overall behavior remained less ductile compared to the steel control specimen.

Overall, the observed agreement between the experimental and numerical crack patterns, along with the consistent identification of the governing flexural failure mode, confirms the capability of the developed finite element model to realistically simulate the cracking and failure behavior of HSC slabs reinforced with steel and CFRP bars.

## Analytical prediction of flexural strength

The analytical comparison was used as a design-assessment framework to evaluate the applicability of existing code provisions to CFRP-reinforced HSC two-way slabs. The comparison between ECP 208–2019^36^ and ACI 440.1R-15^1^ was not intended only to report prediction ratios, but also to identify the level of conservatism and suitability of these provisions for the tested slab configuration.

### Based on ECP 208–2019^36^

**Fig. 16:**
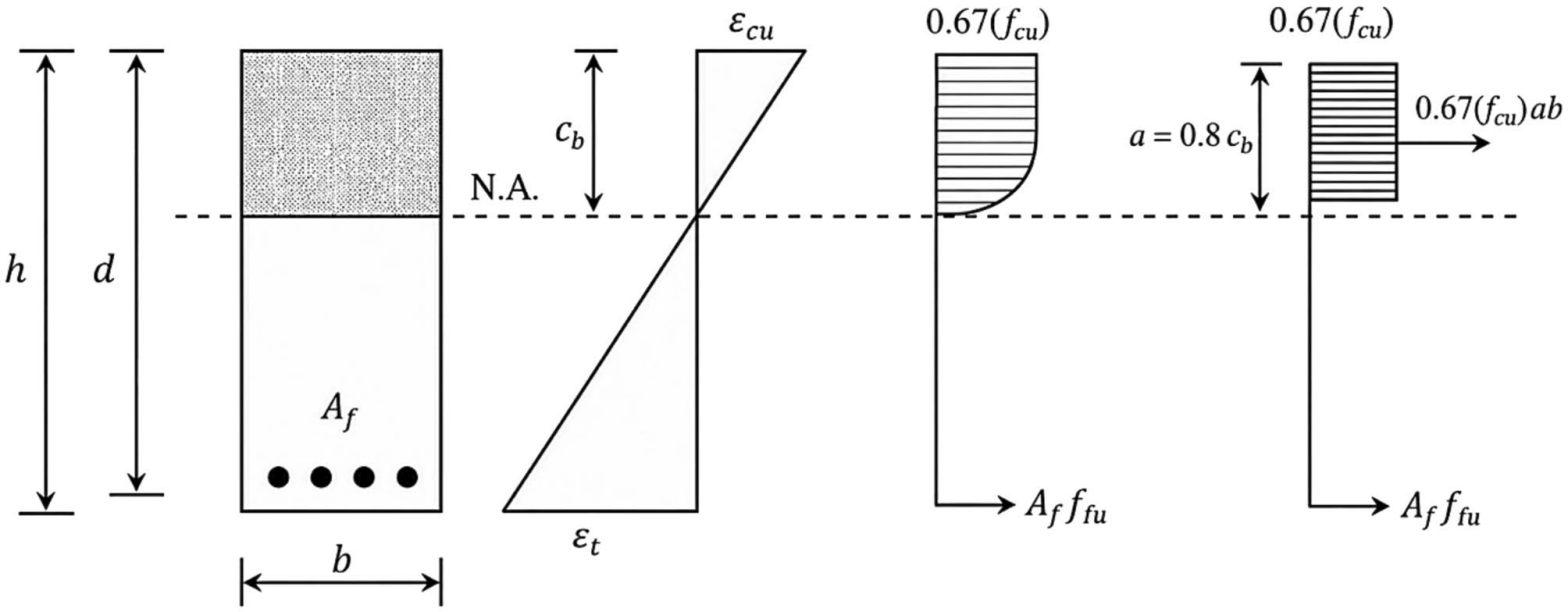
Idealized strain distribution, equivalent stress block, and internal force equilibrium adopted for flexural resistance analysis of CFRP-reinforced slab sections.

The flexural strength of the tested slabs was analytically evaluated according to ECP 208–2019^36^ using strain compatibility and internal force equilibrium, as illustrated in Fig. 16**.** Based on this approach, the concrete compression force and the CFRP tensile force were first determined, and the corresponding flexural moment capacity was then calculated using the following equations:

5 $$C=\frac{0.67{f}_{cu}}{{\gamma}_{c}}ba$$

6 *a = 0.8 c*

7 $${\varepsilon}_{f}={\varepsilon}_{cu}\frac{d-c}{c}$$

8 $${f}_{fe}=\mathrm{min}\left({E}_{f}{\varepsilon}_{f},\frac{{C}_{e}{f}_{fu}}{{\gamma}_{f}}\right)$$

9 $${T}_{f}={A}_{f}{f}_{fe}$$

10 $${M}_{u}={A}_{f}{f}_{fe}\left(d-\frac{a}{2}\right)$$

where *C* is the concrete compression force, ${T}_{f}$ is the tensile force in the CFRP reinforcement, *a* is the depth of the equivalent rectangular stress block, *c* is the neutral axis depth, ${C}_{e}$ is the environmental reduction factor (= 0.8), ${E}_{f}$ is the elastic modulus of CFRP (= 150,000 MPa), ${f}_{cu}$ is the concrete cube compressive strength, ${f}_{fe}$ is the effective stress in the CFRP reinforcement, ${f}_{fu}$ is the ultimate tensile strength of CFRP (= 1400 MPa), ${\gamma}_{c}$ is the partial safety factor for concrete, ${\gamma}_{f}$ is the partial safety factor for CFRP (= 1.3), ${\varepsilon}_{f}$ is the strain in the CFRP reinforcement, ${\varepsilon}_{cu}$ is the ultimate concrete compressive strain(= 0.003), ${A}_{f}$ is the area of CFRP reinforcement, *b* is the slab width, *d* is the effective depth, and ${M}_{u}$ is the nominal moment capacity.

### Based on ACI 440.1R-15^1^

According to ACI 440.1R-15^1^, the nominal flexural strength of the CFRP-reinforced slab section was evaluated using strain compatibility and the equivalent rectangular stress block. The main design equations are expressed as follows:

11 $${M}_{u}\le \phi {M}_{n}$$

12 $${M}_{n}={\rho}_{f}{f}_{f}\left(1-0.59\frac{{\rho}_{f}{f}_{f}}{{f}_{c}{\prime}}\right)b{d}^{2}$$

13 $${f}_{f}=\sqrt{\frac{{\left({E}_{f}{\varepsilon}_{cu}\right)}^{2}}{4}+\frac{0.85{\beta}_{1}{f}_{c}{\prime}}{{\rho}_{f}}{E}_{f}{\varepsilon}_{cu}}-0.5{E}_{f}{\varepsilon}_{cu}\le {f}_{fu}$$

14 $$\phi = 0.55\left| {{\mathrm{for}}} \right|{{\rho }}_{f} \le {{\rho }}_{{fb}} ;\phi = 0.3 + 0.25\frac{{{{\rho }}_{f} }}{{{{\rho }}_{{fb}} }}\left| {{\mathrm{for}}} \right|{{\rho }}_{{fb}} < {{\rho }}_{f} < 1.4{{\rho }}_{{fb}} ;\phi = 0.65\left| {{\mathrm{for}}} \right|{{\rho }}_{f} \ge 1.4{{\rho }}_{{fb}}$$

15 $${\rho}_{f}=\frac{{A}_{f}}{bd}$$

16 $${\rho}_{fb}=0.85{\beta}_{1}\frac{{f}_{c}{\prime}}{{f}_{fu}}\frac{{E}_{f}{\varepsilon}_{cu}}{{E}_{f}{\varepsilon}_{cu}+{f}_{fu}}$$

where M_*u*_ is the required moment, M_*n*_ is the nominal moment capacity, *Φ* is the strength reduction factor,${\rho}_{f}$ is the CFRP reinforcement ratio, ${\rho}_{fb}$ is the balanced CFRP reinforcement ratio, ${f}_{f}$ is the stress in CFRP reinforcement at nominal strength, ${f}_{fu}$ is the ultimate tensile strength of CFRP, ${f}_{c}{\prime}$ is the concrete compressive strength, ${E}_{f}$ is the elastic modulus of CFRP, ${\varepsilon}_{cu}$ is the ultimate concrete compressive strain, ${\beta}_{1}$ : factor is taken as 0.85 for concrete strength $f^{\prime}_{c}$ up to 28 MPa. For strength above 28 MPa, this factor is reduced continuously at a rate of 0.05 per every 7MPa of strength over 28 MPa but isn’t taken less than 0.65, ${A}_{f}$ is the area of CFRP reinforcement, b is the slab width, and d is the effective depth.

### Comparison of results

A comparison was conducted between the experimental results and the analytical predictions obtained from ECP 208–2019^36^ and ACI 440.1R-15^1^, as presented in Table 8. The average analytical-to-experimental ratio was 1.43 for ECP and 1.27 for ACI, indicating that the ACI predictions are in closer agreement with the experimental results. Furthermore, the low standard deviation values indicate a consistent trend across all specimens.

**Table 8:** Comparison between experimental results and analytical predictions from ECP 208–2019^36^ and ACI 440.1R-15^1^.

Slab ID	P_*u, ECP*_ (kN)	P_*u*_*,*_*ACI*_ (kN)	P_*u,ECP*_* /* P_*u,Exp*_	P_*u,ACI*_* /* P_*u,Exp*_
S100-CFRP1	352.08	313.68	1.42	1.27
S100-CFRP2	418.56	372.48	1.49	1.32
S100-CFRP3	483.60	430.08	1.57	1.40
S120-CFRP1	456.00	406.56	1.44	1.29
S120-CFRP2	543.36	484.08	1.31	1.17
S120-CFRP3	629.04	560.16	1.34	1.20
Average	-	-	1.43	1.27
Standard Deviation	-	-	0.09	0.08

Note: P_*u,Ecp*_ is the analytical ultimate load predicted according to ECP 208–2019, while P_*u*_*,*_*ACI*_ is the analytical ultimate load predicted according to ACI 440.1R-15.

## Conclusions

Based on the experimental, numerical, and analytical investigation of two-way high-strength concrete slabs reinforced with CFRP bars, the following conclusions can be drawn:

1. Although S100-Steel and S100-CFRP2 had the same nominal reinforcement ratio of 0.42% and the same slab thickness of 100 mm, they were not mechanically equivalent because steel and CFRP reinforcement have different elastic moduli, tensile strengths, and stress–strain behavior. Mechanical normalization showed that S100-CFRP2 had lower effective reinforcement stiffness but a higher strength-based mechanical reinforcement index. Therefore, the approximately 13% higher ultimate load of S100-CFRP2 should be attributed to the higher tensile capacity of CFRP reinforcement and the post-cracking response of the slab system, rather than to the nominal reinforcement ratio alone.
2. Increasing the CFRP reinforcement ratio improved the structural performance of the slabs. Within Group A, increasing the reinforcement ratio increased the ultimate load by up to 24%, while in Group B the increase reached approximately 48%.
3. As expected from flexural mechanics, increasing the slab thickness from 100 to 120 mm improved the load-carrying capacity of the corresponding CFRP-reinforced slabs, with increases reaching up to 52%. This improvement is attributed to the larger effective depth, flexural rigidity, and internal lever arm.
4. CFRP-reinforced slabs exhibited significantly lower ultimate deflection compared with the steel-reinforced slab, with reductions reaching approximately 50%. This indicates lower deformation capacity and a higher measured global stiffness response under the tested conditions, rather than higher material stiffness of CFRP reinforcement.
5. The steel-reinforced slab showed the highest toughness and deformation capacity, with toughness exceeding that of the CFRP-reinforced slabs by up to 43%. The deformability index of the steel-reinforced slab reached 30.8, compared with values ranging from 7.1 to 19.5 for the CFRP-reinforced slabs.
6. Although CFRP bars have a lower elastic modulus than steel reinforcement, S100-CFRP2 showed higher measured global secant stiffness than S100-Steel. This does not represent a contradiction because the reported secant stiffness is a slab-system parameter evaluated from the load–deflection response at 50% of the ultimate load, not a material stiffness. The higher value is attributed to the higher evaluation load level, the linear-elastic response of CFRP reinforcement, localized cracking, and possible improvement in mechanical interlock due to the ribbed CFRP surface.
7. The nonlinear finite element model developed using ABAQUS demonstrated good agreement with the experimental results. The ratio of experimental to numerical ultimate load ranged from 0.87 to 1.00, with an average of 0.93, while the deflection ratio ranged from 1.02 to 1.12, with an average of 1.07, indicating acceptable prediction accuracy.
8. The numerical model slightly overestimated the first cracking load, with differences ranging from 1.65% to 46.33%. This is mainly attributed to the idealized material properties, perfect bond assumption, and absence of initial micro-cracks in the numerical simulation.
9. The analytical comparison showed that ACI 440.1R-15 provided closer predictions than ECP 208–2019 for the tested CFRP-reinforced slabs. This indicates that the ECP predictions were more conservative for this slab configuration, while the ACI provisions better captured the experimental flexural strength trend.
10. The integrated experimental, numerical, and analytical assessment provides a benchmark and design-assessment framework for CFRP-reinforced HSC two-way slabs. The mechanically normalized steel–CFRP comparison, validated nonlinear finite element model, and ECP/ACI code assessment help clarify the governing flexural mechanisms and the suitability of existing design provisions for this slab configuration.
11. Future studies are recommended to extend the experimental and numerical database of CFRP-reinforced HSC two-way slabs by considering wider ranges of slab thickness, reinforcement ratio, bar surface configuration, support conditions, loading schemes, bond-slip behavior, long-term durability, and hybrid reinforcement systems to improve design recommendations and broaden the applicability of the validated numerical model and analytical assessment framework.

## Study limitations

The present study has several limitations. First, only one steel-reinforced reference slab was included; thus, comparisons with steel are specimen-specific, not generalized material-level assessments. The effects of reinforcement ratio and slab thickness are isolated within the tested envelope (constant thickness groups with varied ratios), but generalization beyond the investigated parameters remains limited.

Second, strain measurements were limited to a single strain gauge installed at mid-span. No full-field deformation techniques (e.g., DIC) were used, and crack development was documented qualitatively through visual observation. These instrumentation limitations restrict local failure mechanism analysis and reduce local model validation strength, though the numerical model (validated globally with ~ 7.2% error) partially compensates.

Third, bond-slip was not modeled (perfect bond assumption). These limitations were considered in interpreting the results and are recommended for future investigation.

## Data Availability

All data generated or analyzed during this study are included in this published article.
